# ADAMTS18 deficiency associates extracellular matrix dysfunction with a higher risk of HER2-positive mammary tumorigenesis and metastasis

**DOI:** 10.1186/s13058-024-01771-3

**Published:** 2024-01-29

**Authors:** Jiahui Nie, Suying Dang, Rui Zhu, Tiantian Lu, Wei Zhang

**Affiliations:** 1 Key Laboratory of Brain Functional Genomics (Ministry of Education and Shanghai), School of Life Science, East China Normal University, 3663 North Zhongshan Road, Shanghai, 200062 China; 2 Department of Biochemistry and Molecular Cell Biology, Shanghai Jiao Tong University School of Medicine, 227 South Chongqing Road, Shanghai, 200025 China

**Keywords:** ADAMTS18, HER2-positive mammary tumorigenesis, Metastasis, Extracellular matrix, Myoepithelial cell

## Abstract

**Background:**

Human epidermal growth factor receptor 2 (HER2)-positive breast cancer accounts for about 20% of all breast cancer cases and is correlated with a high relapse rate and poor prognosis. *ADAMTS18* is proposed as an important functional tumor suppressor gene involved in multiple malignancies, including breast cancer. It functions as an extracellular matrix (ECM) modifier. However, it remains unclear whether ADAMTS18 affects mammary tumorigenesis and malignant progression through its essential ECM regulatory function.

**Methods:**

To elucidate the role of ADAMTS18 in HER2-positive mammary tumorigenesis and metastasis in vivo, we compared the incidence of mammary tumor and metastasis between *Adamts18*-knockout (MMTV)-Her2/ErbB2/Neu^+^ transgenic mice (i.e., *Her2*^*t/w*^*/Adamts18*^−/−^) and *Adamts18*-wildtype (MMTV)-Her2/ErbB2/Neu^+^ transgenic mice (i.e., *Her2*^*t/w*^*/Adamts18*^+/+^). The underlying mechanisms by which ADAMTS18 regulates HER2-positive tumorigenesis and metastasis were investigated by pathology, cell culture, Western blot and immunochemistry.

**Results:**

*Adamts18* mRNA is mainly expressed in myoepithelial cells of the mammary duct. ADAMTS18 deficiency leads to a significantly increased incidence of mammary tumors and metastasis, as well as mammary hyperplasia in mice, over 30 months of observation. The proliferation, migration and invasion capacities of primary *Her2*^*t/w*^*/Adamts18*^−/−^ mammary tumor cells are significantly higher than those of primary *Her2*^*t/w*^*/Adamts18*^+/+^ mammary tumor cells in vitro. At 30 months of age, the expression levels of laminin (LNα5), fibronectin (FN) and type I collagen (ColI) in the mammary glands of *Her2*^*t/w*^*/Adamts18*^−/−^ mice are significantly increased, and the activities of integrin-mediated PI3K/AKT, ERK and JNK signaling pathways are enhanced.

**Conclusions:**

ADAMTS18 deficiency leads to alterations in mammary ECM components (e.g., LNα5, FN, ColI), which are associated with a higher risk of HER2-positive mammary tumorigenesis and metastasis.

**Supplementary Information:**

The online version contains supplementary material available at 10.1186/s13058-024-01771-3.

## Background

Human epidermal growth factor receptor 2 (HER2) is a key mediator for cell proliferation, angiogenesis, survival, and metastasis [1]. The HER2-positive (HER2 +) subtype accounts for about 20% of all breast cancer cases [1–3]. This subtype is correlated with an increased risk of metastasis and poor patient survival, especially in its later stages [3]. However, nearly 50% of HER2 + patients are not benefited from the current HER2-targeted therapies, such as trastuzumab, and nearly 20% of HER2 + patients relapse after treatment [3]. Therefore,identification of key cellular and molecular mechanisms involved in HER2-positive breast cancer may help to develop new disease markers and therapeutic targets.

A large number of studies have shown that extracellular matrix (ECM) plays a crucial role in breast tumorigenesis and metastasis [4–6]. Breast ECM mainly exsits in stroma, including basement membrane (BM) and other ECM molecules in stroma [4]. BM is an amorphous sheet-like structure composed of type IV collagen (ColIV), laminin (LN)-111, LN-332, LN-511, LN-521, proteoglycans, and glycoproteins. Other ECMs in stroma contain type I, II, and III collagen (ColI, II, III), fibronectin (FN), vitronectin, and elastin, which are produced by stromal cells. Disruption of the normal BM, alterations in stroma mechanics, and replacement of normal ECM components by neoplastic ECM components have been shown to promote breast tumorigenesis and metastasis through activation of intracellular signals such as Wnt, PI3K/AKT, ERK and JNK [4–7]. Expression profile of the genes encoding the ECM molecules in stroma and BM have been shown to be more reliable predictors of prognosis in breast cancer patients than tumor epithelial markers [8, 9]. Nonetheless, the key factors that control breast cancer progression by modifying BM and other stromal ECM components remain largely unknown due to the lack of suitable animal models.

The ADAMTSs (a disintegrin and metalloproteinase with thrombospondin motifs) are a group of secreted metalloproteinases with 19 members in humans and are involved in a variety of physiological processes and diseases [10, 11]. Among them, ADAMTS18 has been shown to be essential for the development of several organs, such as epithelial organs [12–17], vascular and neuronal systems [18, 19], and adipose tissue [20]. ADAMTS18 deficiency significantly alters the expression levels and distribution of several ECM molecules in these organs. For example, ADAMTS18 deficiency results in increased levels of LN, ColI, ColIV, and FN in the mammary glands of pubertal mice [15], FN accumulation in developing lacrimal glands and common carotid arteries [17, 18], and bronchial microfibril accumulation in embryonic lungs in mice [13].

*ADAMTS18* has been identified as an important functional tumor suppressor gene involved in multiple carcinomas, including breast cancer [21]. Several studies have shown an association between *ADAMTS18* gene and breast cancer. These findings include: (I) Deletion of chromosome 16q region containing *ADAMTS18* gene is associated with the occurrence and prognosis of breast cancer [22]. (II) *ADAMTS18* expression is reduced in primary breast cancer tissues compared to their adjacent non-cancerous tissues, largely due to promoter methylation [21, 23]. (III) *ADAMTS18* expression is silenced or down-regulated in breast cancer cell lines, and demethylation restores *ADAMTS18* expression in these cells [23]. Furthermore, ectopic expression of ADAMTS18 inhibits migration and invasion of breast cancer cells via AKT and NF-κB signaling pathway, and prevents experimental lung metastasis of breast cancer [23]. These findings suggest that ADAMTS18 deficiency worsens breast cancer. However, there is currently no in vivo evidence from the mouse model of spontaneous mammary cancer showing the effect of ADAMTS18 on mammary tumorigenesis and metastasis through its essential ECM regulatory function.

Mouse mammary tumor virus (MMTV)-Her2/ErbB2/Neu^+^ transgenic mouse model (thereafter named *Her2*^*t/t*^) is a well-characterized mouse model recapitulating the pathological process of mammary tumorigenesis and dissemination [24, 25]. In this study, we developed *Her2*^*t/w*^ transgenic mice with or without ADAMTS18 and showed that ADAMTS18 deficiency associates extracellular matrix alterations (e.g., LNα5, FN, ColI) with a higher risk of HER2-positive mammary tumorigenesis and metastasis.

## Methods

### Animals

*Adamts18* heterozygous mice (*Adamts18*^+/−^) with C57BL6 background were developed in our laboratory [13]. The *Her2*^*t/t*^ transgenic mice (FVB) were generous gifts from Dr. Huang Lei (Shanghai Jiaotong University School of Medicine, Shanghai, China). This FVB-derived transgenic strain developed mammary tumors by overexpressing HER2/Neu oncogene in luminal epithelial cells of the mammary gland under the transcriptional control of the MMTV promoter [24]. The mice used in this study were generated using the following breeding strategy. Homozygous *Her2* transgenic mice (*Her2*^*t/t*^, FVB background) were crossed with heterozygous *Adamts18* gene knockout mice (*Adamts18*^+/−^, C57BL/6 background). All the F1 generation hybrid mice (i.e., *Her2*^*t/w*^*/Adamts18*^+*/*+^, and *Her2*^*t/w*^*/Adamts18*^+/−^) had a background of 1/2 FVB and 1/2 C57BL6. F1 mice were then crossbred with *Adamts18*^+*/*+^ and *Adamts18*^*−/−*^ mice (C57BL/6 background). The genetic background of most F2 generation hybrid mice was 1/4 FVB and 3/4 C57BL6. Specifically, the F1 mice with *Her2*^*t/w*^*/Adamts18*^+*/*+^ genotype were crossbred with *Adamts18*^+*/*+^ mice to obtain the *Her2*^*t/w*^*/Adamts18*^+*/*+^ mice (1/2 of the offsprings). The F1 mice with *Her2*^*t/w*^*/Adamts18*^+/−^ genotype were crossbred with *Adamts18*^*−/−*^ mice to obtain the *Her2*^*t/w*^*/Adamts18*^*−/−*^ mice (1/4 of the offsprings). The above breeding strategy for obtaining *Her2*^*t/w*^*/Adamts18*^+*/*+^ or *Her2*^*t/w*^*/Adamts18*^*−/−*^ mice ensured that the experimental and control mice were similar with respect to the genetic contributions of FVB and C57BL6 (Additional file 1: Fig. S1A). Mice, including *Her2*^t/t^ mice, *Her2*^*t/w*^*/Adamts18*^+/+^ mice, and *Her2*^*t/w*^*/Adamts18*^−/−^ mice, were examined weekly for palpable mammary tumors until 30 months of age. Tumor size was measured with calipers and the survival rate was monitored daily. All procedures for animal experiments were approved by the Institutional Animal Care and Use Committee of East China Normal University in accordance with the ARRIVE (Animal Research: Reporting of In Vivo Experiments) guidelines.

### Pathological analysis and RNA in situ hybridization

At the end of the in vivo experiments, a complete autopsy was performed. Tissue samples were collected, fixed in 10% neutral formalin, embedded into paraffin blocks, then sectioned and stained for hematoxylin and eosin (HE) and/or immunohistochemistry (IHC)/immunofluorescence (IF), as well as RNA in situ hybridization (ISH). For IHC and IF assays, slides were incubated with indicated primary antibodies (Additional file 1: Table S1), followed by secondary antibodies and imaged under a microscope as previously described in detail [13, 16–18]. For RNA ISH, target mRNA in mouse mammary gland was detected using a specific *Adamts18* probe, as described previously [13]. For the whole-mount mammary gland assay, mammary glands were extracted from virgin females and flattened on a slide. The slides were air-dried for 5 min and fixed with 4% paraformaldehyde. Mammary tissues were transferred to 100% acetone (3 times) to remove fat, and then to 100% and 90% ethyl alcohol (EtOH). The mammary tissues were stained in hematoxylin for 3 h, rinsed and incubated in 50% EtOH, then transferred to 70, 95, 100% EtOH (1 h each) and xylene (2 times), and preserved in xylene.

### Primary culture of mammary tumor cells

Primary isolation and culture of mouse mammary tumors were performed according to the previous method [26]. In briefly, the tumor growing subcutaneously near the fat pad of the mammary gland was exposed under aseptic conditions and the mammary tumor was carefully removed with scissors. Part of the tumor tissue was used for cell separation, and the rest was frozen or fixed for other experimental analysis. The mammary tumor was placed in a petri dish on ice, a small amount of medium was added, the tissue was chopped up with a blade, and the medium was added to ensure that the tissue does not dry out. Transfer the pieces into a 15 ml tube. DMEM medium containing 10% FCS plus 1 mg/ml collagenase D was added to the chopped tissue. The tissue was digested in a rotating Hybrid oven at 37 °C for at least 1 h. After digestion, medium was added to inactivate digestive enzymes. The red blood cells were removed by continuous suspension and centrifugation with lysis buffers. During passage, the fibroblasts were removed by a short incubation with trypsin. The primary tumor cells were cultured in medium containing 10% FBS, 10 ng/ml EGF, and 5 μg/ml bovine insulin for 2–4 generations and used for the follow-up experiments.

### Cell viability, wound healing, and invasion assay

Cell viability assay, wound healing assay, and migration and invasion assay were performed as described previously [23]. For cell viability assay, mouse mammary tumor cells of different genotypes were cultured in 96-well plates for 12 h. Cell Counting Kit-8 (CCK-8, Beyotime, Shanghai, China) was used for further measurement. For wound healing assay, cells were cultured overnight, then the medium was discarded and a mechanical wound was created with a pipette tip perpendicular to the cell plane. Fresh serum-free medium was then added, and images were taken at different time points (0, 6, 12 h) to measure and quantify the distance between wound edges. For migration and invasion assay, the cells migrated to the lower side of the membrane were stained with 0.1% crystal violet. The cells were then counted in five microscope fields and averaged.

### Quantitative real-time RT-PCR, Sandwich ELISA, and Western blotting analysis

Quantitative Real-Time RT-PCR (qRT-PCR), Sandwich ELISA, and Western blotting analysis were performed essentially as previously described in detail [13, 16–18]. Briefly, for qRT-PCR, total RNA was extracted from mouse mammary tissues and qRT-PCR was performed using the StepOnePlus real-time PCR system. The sequences of primers used in qRT-PCR have been described previously [13, 16–18]. For Sandwich ELISA, mouse mammary tissues were dissected, weighted, and homogenized. Concentrations of LN, ColI, and ColIV were determined according to the manufacturer’s instructions of the ELISA kit (LYBD Bio-Technique Co, Ltd, Beijing, China). For Western blotting, mouse mammary tissues were homogenized and protein concentration was determined by BCA assay reagent (Pierce, Rockford, IL, USA). The proteins were separated by SDS–PAGE and then transferred to polyvinylidene difluoride (PVDF) membrane. After incubation with primary and secondary antibodies, immunoreactive bands were visualized using enhanced chemiluminescence (ECL) and qualified using ImageJ software, as described previously [13, 16–18].

### Patient samples and data source

HER2-positive breast cancer-associated GEO datasets (GSE191230) were downloaded from GEO database (https://www.ncbi.nlm.nih.gov/gds), which contains RNA expression of tumor from 13 treatment-naïve HER2 + breast tumors and 7 distant metastases of HER2 + tumors relapsed posttrastuzumab treatment. RNA expression was quantified with transcripts per kilobase of exon model per million mapped reads (TPM). Analysis of de-identified data in the GEO database does not require the approval of institutional review board and informed consent. Due to the normalization of data, data were not processed in this study.

### Statistics

Data were analyzed using Prism version 8 (GraphPad, La Jolla, CA, USA) and were shown as mean ± SD using the two tailed Student’s *t* test (for comparison between the two groups), or one-way ANOVA (for comparison between the three groups), or Log-rank (Mantel-Cox) test for mouse survival experiments. Tumor incidence between the two groups was tested using chi-squared analysis. Statistical significance was accepted at *P* < 0.05.

## Results

### *Adamts18* mRNA is expressed in the myoepithelial cells of mammary ducts

**Fig. 1:**
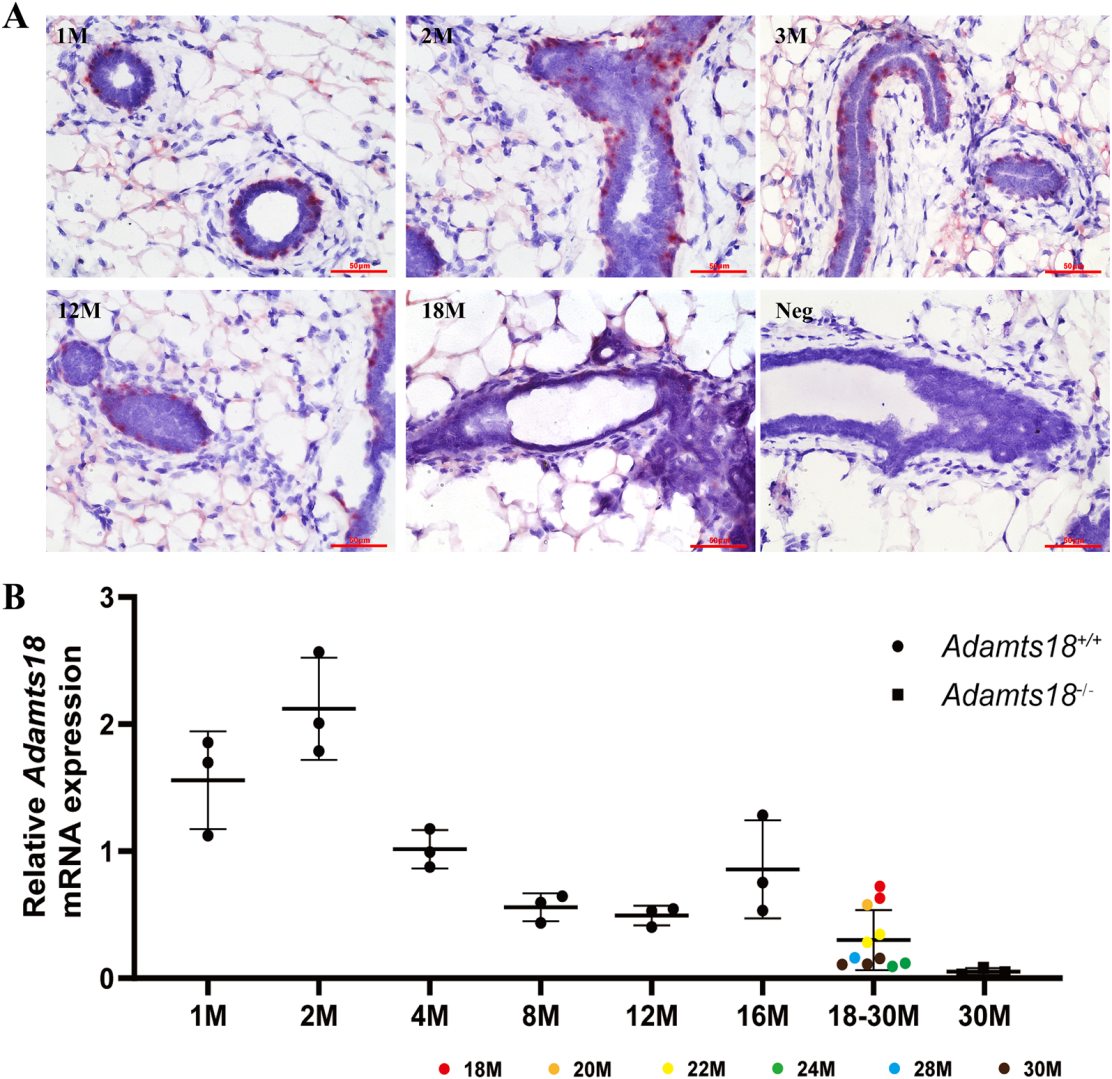
Expression of *Adamts18* mRNA in mouse mammary gland. **(A)** In situ hybridization analysis of *Adamts18* mRNA in mammary gland of mice. In situ hybridization–positive signals appear as pink dots in cells. Scale bar = 50 μm. M, month. Neg, negative probe hybridization of mammary gland as negative control. **(B)** The expression of *Adamts18* mRNA in mammary gland of mice aged from 1 to 30 months was analyzed by qRT-PCR. The relative quantity of *Adamts18* mRNA was normalized to that of the housekeeping gene *Gapdh* using the ΔΔCt method. Each dot represents an individual. Data are expressed as mean ± SD

The distribution of *Adamts18* mRNA in mouse mammary glands was determined by in situ hybridization [10]. The results showed that *Adamts18* was mainly expressed in the myoepithelial cells of mammary ducts at different stages, including pubertal stage (1-month, M), adult stage (2M, 3M, and 12M), and elderly stage (18M) (Fig. 1A). *Adamts18* mRNA levels in mammary tissues of female virgin mice aged from 1 to 30 months were also detected by qRT-PCR. The results showed that *Adamts18* expression was highest at 2 months of age, and then decreased in adult and maintained at low level in elderly mammary tissues of virgin females (Fig. 1B).

### ADAMTS18 deficiency increases the risk of HER2-positive mammary tumorigenesis and metastasis

The progression of primary mammary epithelial cells to a malignant phenotype involves multiple genetic events, including activation of oncogenes and inactivation of specific tumor suppressor genes. To investigate the association of ADAMTS18 with HER2-positive breast cancer, we developed *Her2*^*t/w*^*/Adamts18*^+/+^ and *Her2*^t/w^*/Adamts18*^−/−^ mice with C57BL/6-FVB mixed background for subsequent experiments (Additional file 1: Fig. S1A). Female virgin mice were palpated weekly to detect the presence of spontaneous mammary tumors until 30 months of age. There was no significant difference in mean body weight between *Her2*^t/w^*/Adamts18*^+/+^ mice and *Her2*^t/w^*/Adamts18*^−/−^ mice during the 30-month observation period (Additional file 1: Fig. S1B). The median survival time of *Her2*^*t/w*^*/Adamts18*^−/−^ mice was shorter than that of *Her2*^*t/w*^*/Adamts18*^+/+^ mice (26 months vs 28.8 months, *P* = 0.0001) (Additional file 1: Fig. S1C), but the survival curve does not distinguish between natural and tumor-related deaths as mice both with and without visible tumors began to die after 26 months.

**Fig. 2:**
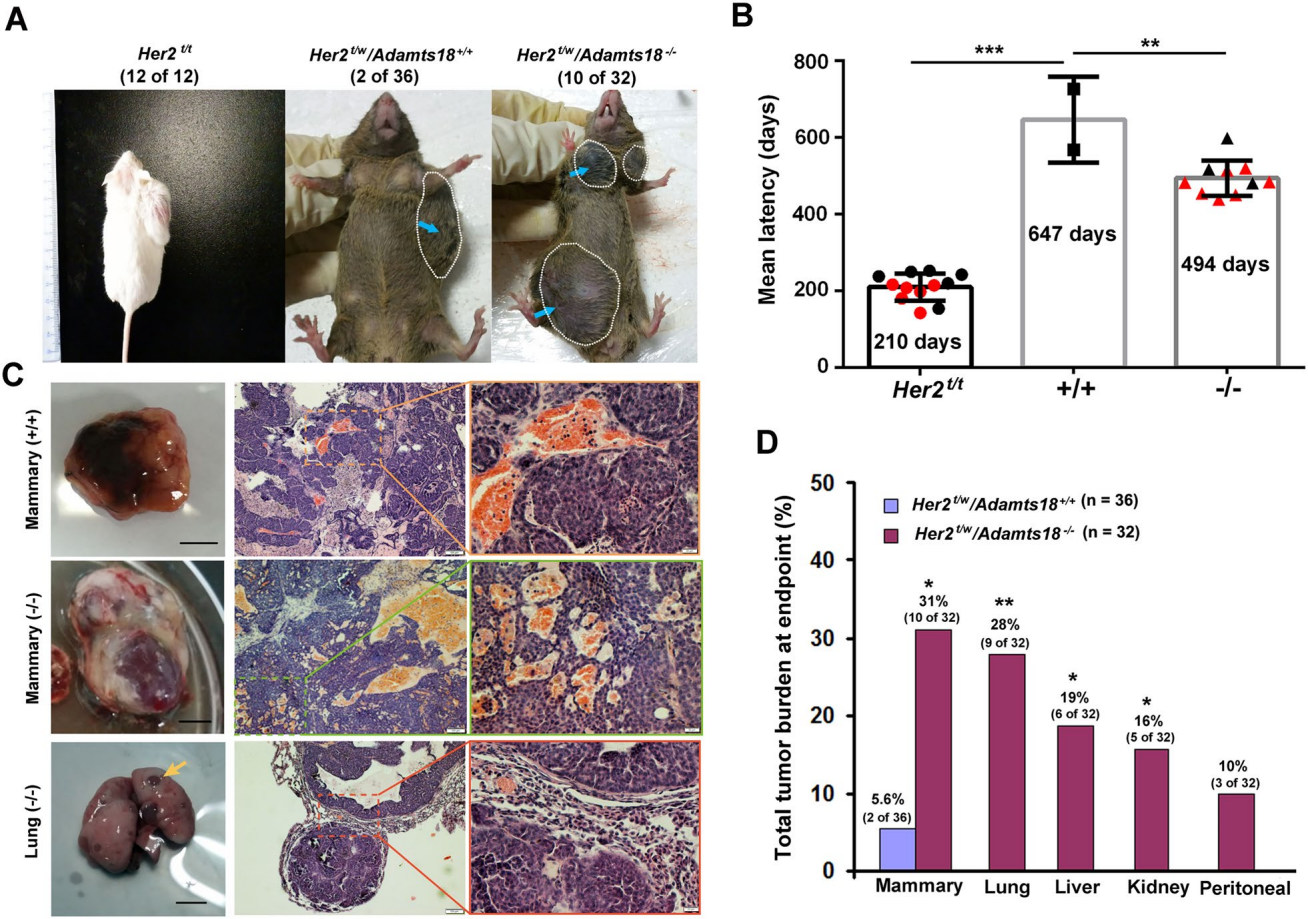
ADAMTS18 deficiency increases the risk of HER2-positive mammary tumorigenesis and metastasis. (**A**) Representative images of spontaneous mammary tumors and the incidence of macroscopic mammary tumor cases in *Her2*^*t/t*^ mice with FVB background (12 of 12, 100%), and *Her2*^*t/w*^*/Adamts18*^+/+^ mice (2 of 36, ~ 5.6%) and *Her2*^*t/w*^*/Adamts18*^−/−^ mice (10 of 32, ~ 31%) with FVB-C57BL/6 mixed background. (**B**) The mean latency of spontaneous mammary tumors in tumor-bearing mice. Red represents the formation of multiple tumors in an individual mouse. Each dot, square, or triangle represents an individual. (**C**) Representative images and HE staining of mammary tumors and lung metastasis from the indicated genotypes. Brown arrow, lung tumor. Scale bars, 20 and 100 μm, respectively. In *Her2*^*t/w*^/*Adamts18*^*-/-*^ mice (middle and lower panels), tumor type in the lungs was consistent with the primary site of the mammary tumors. (**D**) The incidence of tumors in distant organs of *Her2*^*t/w*^*/Adamts18*^+/+^ mice (n = 36) and *Her2*^*t/w*^*/Adamts18*^−/−^ mice (n = 32). *p < 0.05; **p < 0.01; ***p < 0.001; *p* values of tumor incidence between two groups were determined through chi-squared analysis

Due to halve *Her2* expression and with FVB-C57BL/6 mixed background, both *Her2*^*t/w*^*/Adamts18*^−/−^ mice (10 of 32, ~ 31%) and *Her2*^*t/w*^*/Adamts18*^+/+^ mice (2 of 36, ~ 5.6%) showed significantly lower incidence of spontaneous mammary tumors when compared with *Her2*^*t/t*^ mice (12 of 12, 100%) (*P* < 0.001) (Fig. 2A). Nevertheless, the incidence of spontaneous mammary tumors was significantly higher in *Her2*^*t/w*^*/Adamts18*^−/−^ mice than in *Her2*^*t/w*^*/Adamts18*^+/+^ mice (31% vs 5.6%, *P* = 0.01). Among the 10 tumor-bearing *Her2*^*t/w*^*/Adamts18*^−/−^ mice, 7 developed more than two mammary tumors, while only one mammary tumor was detected in each of the 2 tumor-bearing *Her2*^*t/w*^*/Adamts18*^+/+^ mice (Additional file 1: Fig. S2). In these tumor-bearing mice, spontaneous tumors appeared significantly earlier in *Her2*^*t/w*^*/Adamts18*^−/−^ mice than in *Her2*^*t/w*^*/Adamts18*^+/+^ mice. The mean latency for mammary tumor development was 210 days in *Her2*^*t/t*^ mice, 494 days in *Her2*^*t/w*^*/Adamts18*^−/−^ mice, and 647 days in *Her2*^*t/w*^*/Adamts18*^+/+^ mice, respectively (Fig. 2B). In addition, the mammary tumor-bearing *Her2*^*t/w*^*/Adamts18*^−/−^ mice exhibited significant lung metastases when compared with *Her2*^t/w^*/Adamts18*^+/+^ mice (-/- vs. + / + : 9 of 32, ~ 28% vs. 0 of 36, 0%; *P* = 0.0022) (Fig. 2C and D). These tumor-bearing *Her2*^t/w^*/Adamts18*^−/−^ mice also developed metastases in the liver (6 of 32, ~ 19%, *P* = 0.0219), kidney (5 of 32, ~ 16%, *P* = 0.0457), and peritoneal cavity (3 of 32, ~ 10%, *P* = 0.198) when compared with *Her2*^*t/w*^*/Adamts18*^+/+^ mice (Additional file 1: Fig. S3). Collectively, the 10 mammary tumor-bearing *Her2*^*t/w*^*/Adamts18*^−/−^ mice were all accompanied by metastases, while the 2 *Her2*^*t/w*^*/Adamts18*^+/+^ mice exhibited only primary mammary tumors (Additional file 1: Table S2), suggesting that ADAMTS18 deficiency increases the risk of HER2-positive spontaneous mammary metastasis.

### ***Her2***^***t/w***^***/Adamts18***^−/−^ mammary tumor cells show increased proliferation, migration, and invasion capacity in vitro

**Fig. 3:**
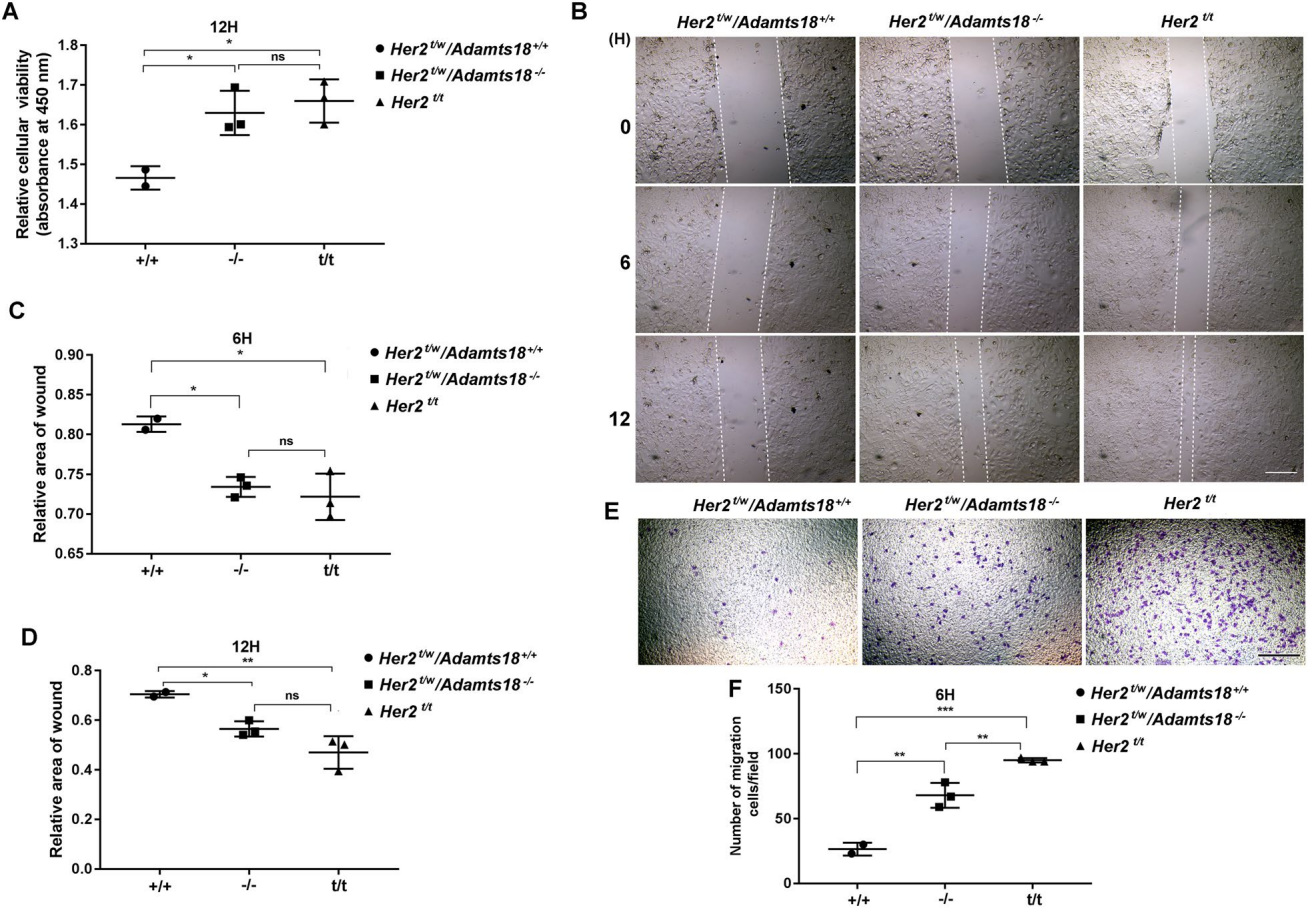
Increased proliferation, migration, and invasion capacity in *Her2*^*t/w*^*/Adamts18*^−/−^ mammary tumor cells. **(A)** Counting kit-8 (CCK8) assay for cell proliferation of mammary tumor cells after 12-h (h) culture. **(B**–**D)** Migration of mammary tumors by scratch wound healing assays. Representative images of migrated mammary tumors in each group are shown (**B**). The scratch healing ratio was obtained by comparing the scratch area of 6 h and 12 h with that of 0 h. A smaller ratio indicates faster cell migration (healing) (**C** and **D**). Scale bar = 50 μm. (**E**–**F**) Transwell assay of invasive ability of mammary tumors. Representative images of migrated mammary tumors in each group are shown (**E**). Scale bar = 200 μm. Cells at the lower surface of the transwell chamber were counted (**F**). Data are expressed as mean ± SD from *Her2*^*t/w*^*/Adamts18*^+/+^ mammary tumors (n = 2), *Her2*^*t/w*^*/Adamts18*^−/−^ mammary tumors (n = 3), and *Her2*^*t/t*^ mammary tumors (n = 3). *p < 0.05; **p < 0.01; ***p < 0.001; ns, not significant; one-way ANOVA

To understand why *Her2*^*t/w*^*/Adamts18*^−/−^ mice are more likely to form tumors and migrate than *Her2*^*t/w*^*/Adamts18*^+/+^ mice, we compared the proliferation, migration, and invasion capacity of primary tumor cells isolated from *Her2*^*t/w*^*/Adamts18*^−/−^ mammary tumors, *Her2*^*t/w*^*/Adamts18*^+/+^ mammary tumors, and *Her2*^t/t^ mammary tumors. At 12 h, *Her2*^*t/t*^ and *Her2*^*t/w*^*/Adamts18*^−/−^ mammary tumor cells showed significantly increased proliferation when compared with *Her2*^*t/w*^*/Adamts18*^+/+^ mammary tumor cells (Fig. 3A). Scratch would healing assays showed that the migration capacity of *Her2*^*t/t*^ and *Her2*^*t/w*^*/Adamts18*^−/−^ mammary tumor cells was significant increased compared with *Her2*^*t/w*^*/Adamts18*^+/+^ mammary tumor cells at 6 h and 12 h, respectively (Fig. 3B-D). Transwell assays showed that the invasion of *Her2*^*t/t*^ and *Her2*^*t/w*^*/Adamts18*^−/−^ mammary tumor cells was significantly increased compared with *Her2*^*t/w*^*/Adamts18*^+/+^ mammary tumor cells at 6 h (Fig. 3E and F).

### ADAMTS18 deficiency promotes mammary hyperplasia

**Fig. 4:**
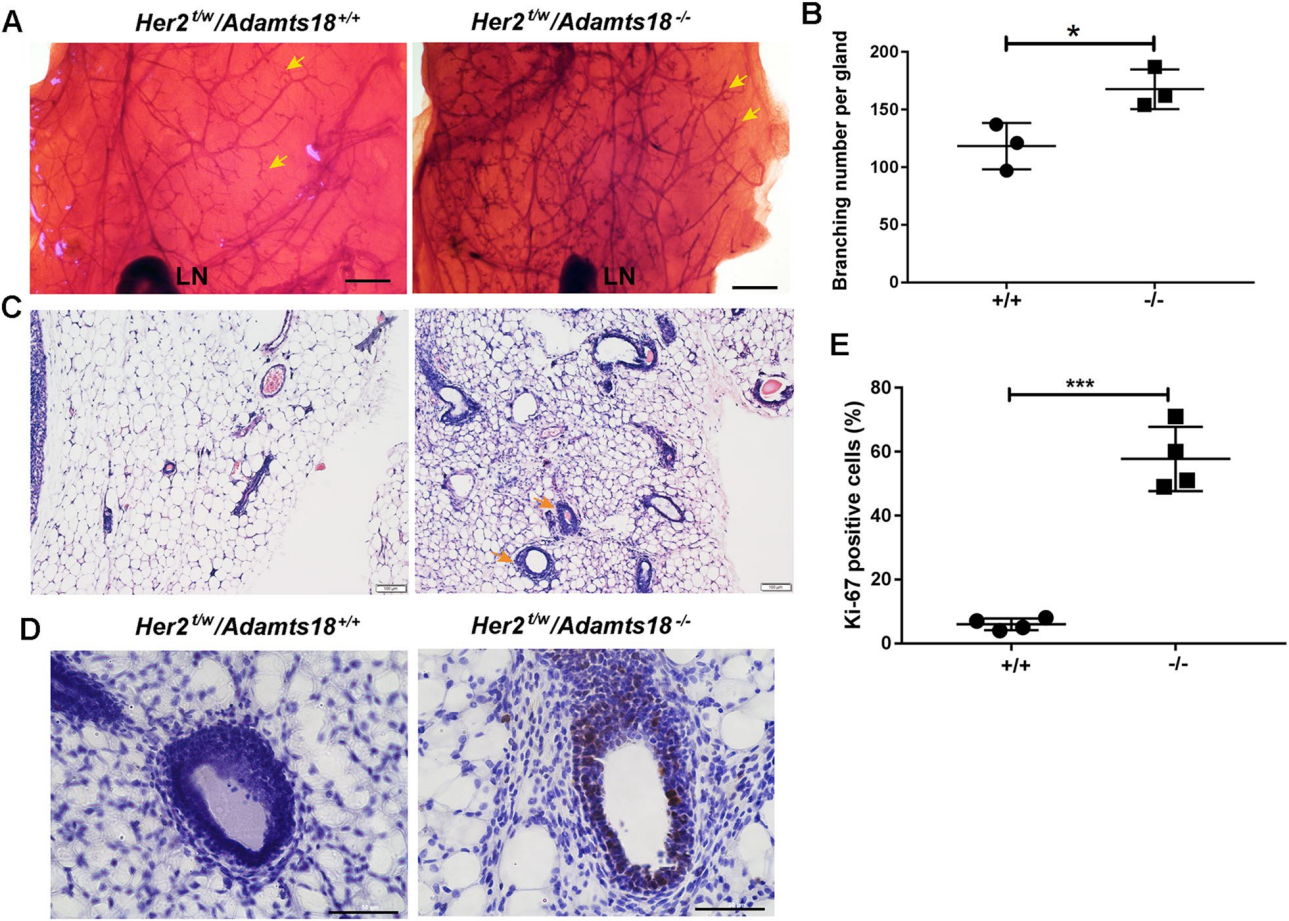
ADAMTS18 deficiency promotes mammary hyperplasia. (**A**) Whole-mount preparation of mammary glands from the indicated genotypes at 30 months of age. Scale bars = 500 μm. Arrowheads indicate branches and terminal end buds. LN, lymph node. (**B**) Quantification of the branch point in the two genotypes indicated in B (n = 3/group). (**C**) Representative H&E-stained cross-sections of mammary glands from the indicated genotypes. Arrowheads indicate thickened tubules. Scale bars = 100 μm. (**D**) Representative immunohistochemical staining of cross-sections of mammary glands from the indicated genotypes using Ki-67 antibody. Scale bars = 50 μm. (**E**) Quantification of Ki-67-positive cells (n = 4/group). Data are expressed as mean ± SD. *p < 0.05; ***p < 0.001; Student’s *t*-test, two tailed

The development of breast cancer is a multi-step process that includes hyperplasia, precancerous lesions, carcinoma in situ, and metastasis. We then characterized the mammary glands of 30-month-old *Her2*^t/w^*/Adamts18*^−/−^ and *Her2*^t/w^*/Adamts18*^+/+^ mice without macroscopic tumors to determine whether ADAMTS18 has an effect on mammary hyperplasia. When compared with those of *Her2*^*t/w*^*/Adamts18*^+/+^ mice, the mammary ducts of *Her2*^*t/w*^*/Adamts18*^−/−^ mice were disordered with an increased number of branches (Fig. 4A and B). Histological analysis showed that the transverse section of the lateral ductal branch of *Her2*^*t/w*^*/Adamts18*^−/−^ mice was thickened (Fig. 4C). The number of Ki-67-positive mammary epithelial cells in the mammary ducts of 30-month-old *Her2*^*t/w*^*/Adamts18*^−/−^ mice was significantly increased (Fig. 4D and E). We further investigated the earlier proliferation of mammary epithelial cells in mice with different genotypes. The results showed that at 10 months of age, Ki-67 positive signals were significantly increased in the mammary ducts of *Her2*^*t/w*^*/Adamts18*^*−/−*^ mice compared with those in *Her2*^*t/w*^*/Adamts18*^+*/*+^ mice (Additional file 1: Figure S4). Together, these results suggest that ADAMTS18 deficiency promotes mammary hyperplasia.

### ADAMTS18 deficiency increases the activity of ERK and PI3K/AKT signaling pathways

**Fig. 5:**
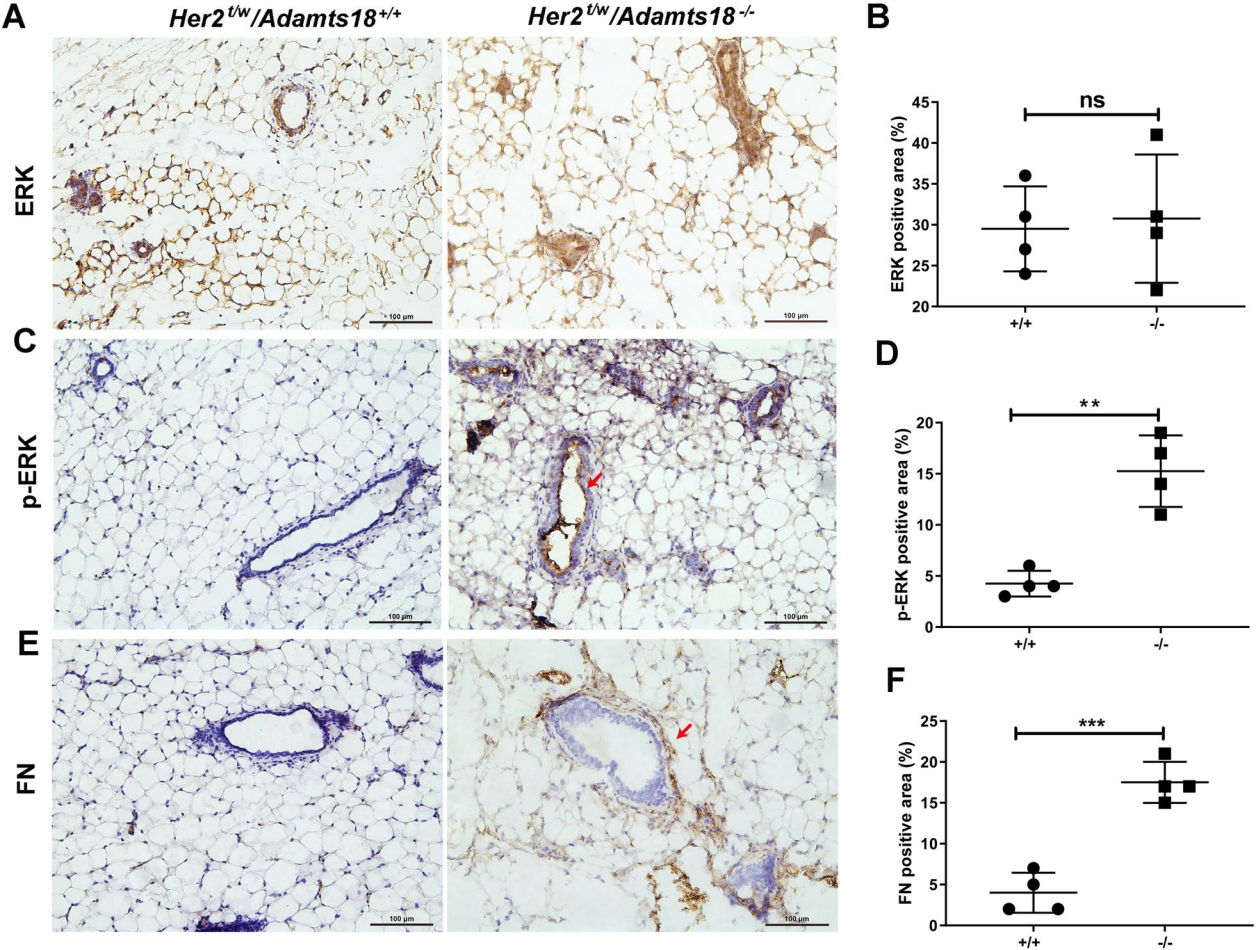
Enhanced ERK1/2 activity in mammary epithelium of 30-month-old *Her2*^*t/w*^*/Adamts18*^−/−^ mice. (**A**, **C**, **E**) Representative immunochemistry staining of total ERK (**A**), Thr202/Tyr204-phosphorylated ERK (**C**), and fibronectin (FN) (**E**) in mammary epithelium. Scale bar, 100 μm. (**B**, **D**, **F**) Quantification of ERK (**B**), pERK (**D**), and FN-positive areas (**F**) was performed with Image Pro Plus. Each dot or square represents an individual. Data are expressed as mean ± SD (n = 4). **p < 0.01; ***p < 0.001; ns, not significant; Student’s *t*-test, two tailed

HER2 is mainly involved in RAS-RAF-MEK-ERK pathway for cell proliferation and PI3K-AKT-mTOR pathway for cell survival [27, 28]. To determine whether ADAMTS18 deficiency synergistically strengthens the HER2 downstream signaling pathway, we examined the activity of ERK signaling pathway in the mammary epithelium of 30-month-old mice with different genotypes. Immunohistochemical analysis showed that the total ERK levels in *Her2*^t/w^*/Adamts18*^+/+^ and *Her2*^t/w^*/Adamts18*^−/−^ mammary glands were comparable (Fig. 5A and B). However, the levels of Thr202/Tyr204-phosphorylated ERK were significantly increased in mammary epithelial cells of *Her2*^t/w^*/Adamts18*^−/−^ mice (Fig. 5C and D). Considering that fibronectin is one of the important indicators involved in epithelial-mesenchymal transitions (EMT) process, and its effect on EMT is mediated by ERK kinase pathway, we then detected the expression level of fibronectin in mammary epithelium of mice with different genotypes. The results showed that the expression of fibronectin in the mammary epithelium of *Her2*^*t/w*^*/Adamts18*^*−/−*^ mice was significantly higher than that of *Her2*^*t/w*^*/Adamts18*^+*/*+^ mice (Fig. 5E and F). Likewise, Western blot results showed that the levels of p-ERK1/2 (Thr202/Tyr204) and p-AKT (Ser-473) were significantly higher in *Her2*^*t/w*^*/Adamts18*^−/−^ mammary glands than those in *Her2*^*t/w*^*/Adamts18*^+/+^ mammary glands (Additional file 1: Fig. S5). The level of E-cadherin (epithelial markers) in *Her2*^*t/w*^*/Adamts18*^−/−^ mammary glands was significantly lower than that of *Her2*^*t/w*^*/Adamts18*^+/+^ mammary glands, while the levels of N-cadherin and fibronectin (mesenchymal markers) were significantly higher in *Her2*^*t/w*^*/Adamts18*^−/−^ mammary glands than those in *Her2*^*t/w*^*/Adamts18*^+/+^ mammary glands (Additional file 1: Fig. S5).

### ADAMTS18 deficiency causes alterations of mammary ECM

**Fig. 6:**
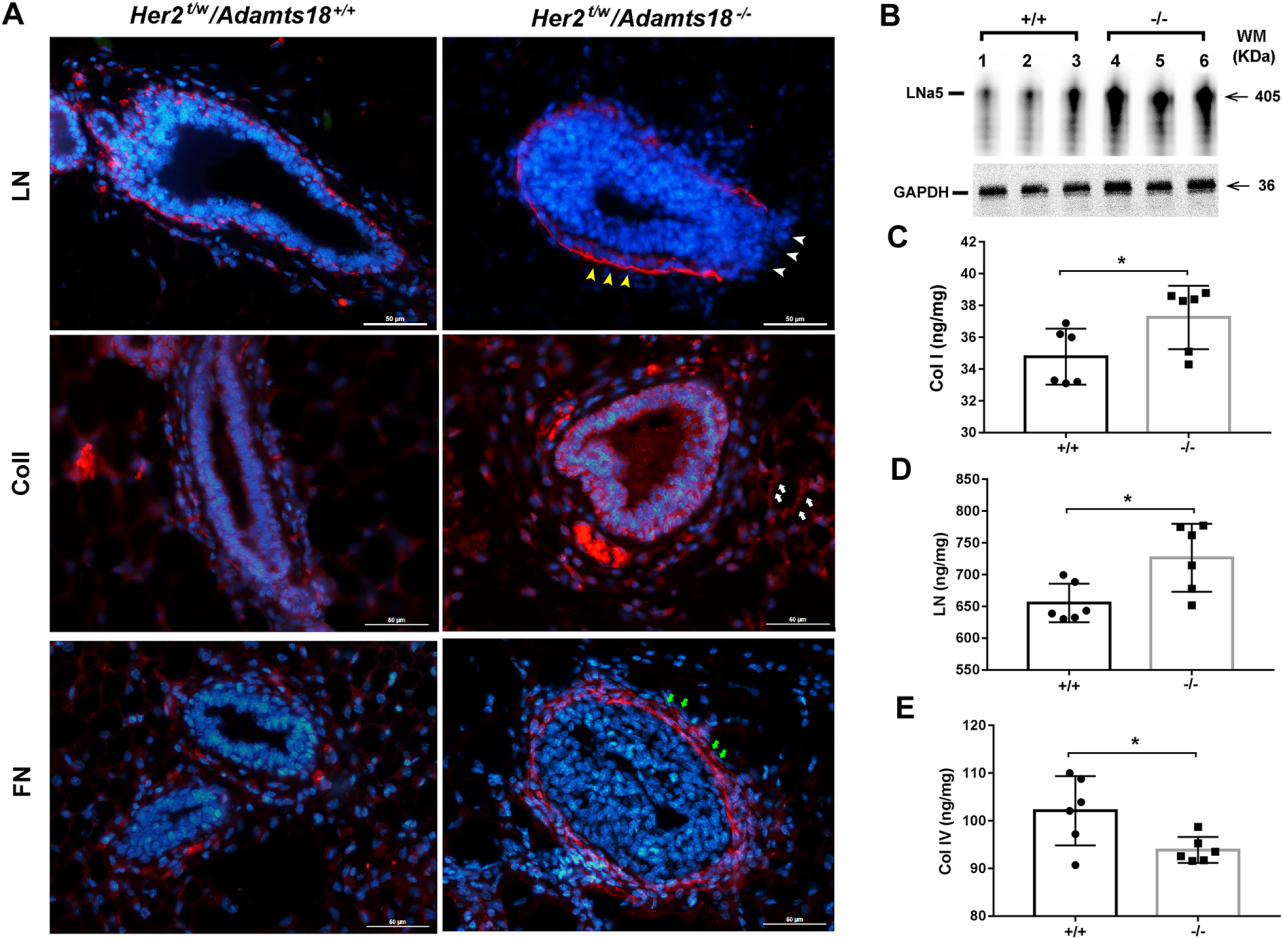
Expression and distribution of extracellular matrix molecules in mammary glands of 30-month-old mice with different genotypes. (**A**) Representative immunofluorescent staining of laminin (LN), collagen I (ColI), and fibronectin (FN) in the mammary glands of mice with different genotypes (30-month-old *Her2*^*t/w*^*/Adamts18*^+/+^ and *Her2*^*t/w*^*/Adamts18*^−/−^ mice). Blue, 4',6-diamidino-2-phenylindole (DAPI); white short arrow, fractured laminin; yellow short arrow, local accumulation of laminin; white arrow, Col I distributed in stroma of mammary glands; Green arrow, fibronectin distributed in BM of mammary glands. Scale bars = 50 μm. (**B**) Western blot analysis of the protein levels of LNα5 in mammary glands of mice (n = 3/group). (**C-E**) Levels of ColI (**C**), LN (**D**), and ColIV (**E**) measured by sandwich enzyme-linked immunosorbent assay (n = 6/group). Data are expressed as mean ± SD (n = 6). *p < 0.05; Student’s *t*-test, two tailed

To determine whether increased ERK1/2 and AKT signaling activity is associated with ECM alterations caused by ADAMTS18 deficiency, we examined the expression and distribution of ECM molecules and epithelial cell receptors in the mammary glands of two genotypes of mice at 30 months of age. qRT-PCR results showed that the transcription levels of *Fn*, *LNα5*, *Col1a1*, *Integrin (Itg)α3*, *Itgα5* and *Itgβ1* were significantly increased in the mammary glands of *Her2*^*t/w*^*/Adamts18*^−/−^ mice at 30 months of age (Additional file 1: Table S3). The expression levels of *Lnα1, β1, β2, β3, γ1, γ2, Itgβ3*, *Dag1, Nid1, Vcan,* and *Ddr1* were comparable between the two genotypes. IHF analysis showed that LN in the *Her2*^*t/w*^*/Adamts18*^+/+^ BM was distributed evenly and continuously, while LN in the *Her2*^*t/w*^*/Adamts18*^−/−^ BM was distributed with local intensification and fragmentation (Fig. 6A). Fibrillar ColI was rarely distributed in the *Her2*^*t/w*^*/Adamts18*^+/+^ mammary stroma, while it was widely distributed in the *Her2*^*t/w*^*/Adamts18*^−/−^ mammary stroma. Compared with the low level of FN distribution in *Her2*^*t/w*^*/Adamts18*^+/+^ BM, FN was abundantly distributed in *Her2*^*t/w*^*/Adamts18*^−/−^ BM. Western blot results showed that the levels of LNα5 were significantly increased in *Her2*^*t/w*^*/Adamts18*^−/−^ mammary glands (Fig. 6B). Sandwich ELISA results showed that the levels of ColI and LN in *Her2*^*t/w*^*/Adamts18*^−/−^ mammary glands were significantly higher than those in *Her2*^*t/w*^*/Adamts18*^+/+^ mammary glands, and the ColIV level in *Her2*^*t/w*^*/Adamts18*^−/−^ mammary glands was significantly lower than that in *Her2*^*t/w*^*/Adamts18*^+/+^ mammary glands (Fig. 6C–E).

**Fig. 7:**
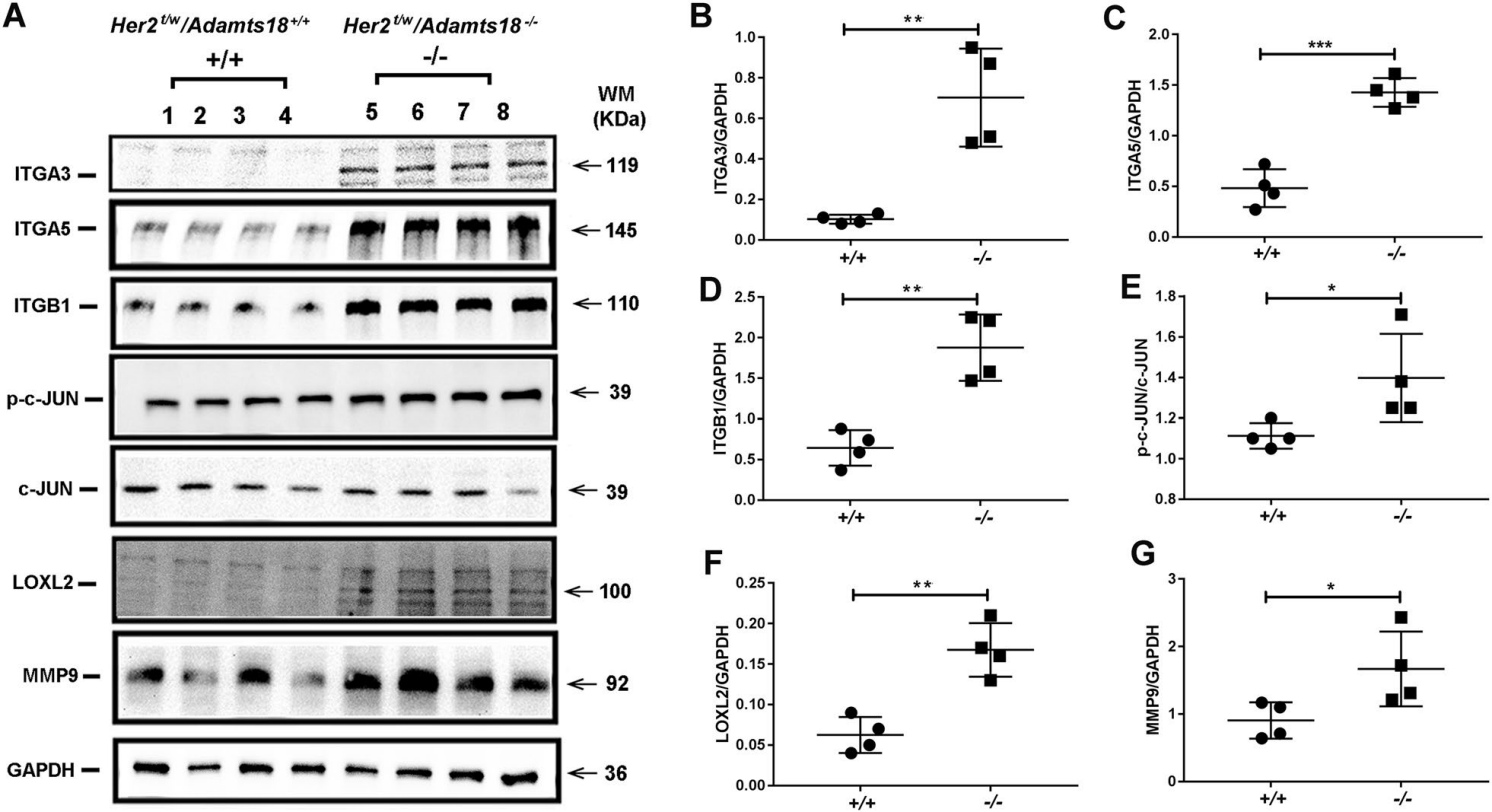
Analysis of the protein levels of integrinα3 (ITGA3), α5 (ITGA5), β1(ITGB1), c-JUN, phosphorylated c-JUN (p–c-JUN), LOXL2 and MMP9 in mammary glands of 30-month-old mice. **(A)** Representative Western blot images of different proteins from the indicated genotypes (30-month-old *Her2*^*t/w*^*/Adamts18*^+/+^ and *Her2*^*t/w*^*/Adamts18*^−/−^ mice). **(B-G)** Relative expression levels of the proteins are represented as p–c-JUN/c-JUN or protein/GAPDH. Each dot or square represents an individual. Data are expressed as mean ± SD (n = 4). *p < 0.05; **p < 0.01; ***p < 0.001; Student’s *t*-test, two tailed

Western blot results showed that the protein levels of ITGα3, ITGα5, ITGβ1, phosphorylated c-JUN, and LOXL2 in *Her2*^*t/w*^*/Adamts18*^−/−^ mammary glands were significantly higher than those in *Her2*^*t/w*^*/Adamts18*^+/+^ mammary glands (Fig. 7A–F). We also examined the expression of MMP2 and MMP9, as they play a key role in the degradation of the BM. The level of MMP9 in mammary glands of *Her2*^*t/w*^*/Adamts18*^−/−^ mice at 30 months of age was significantly higher than that of *Her2*^*t/w*^*/Adamts18*^+/+^ mice (Fig. 7G). There were no significant differences in MMP2 and TIMP-1 levels between *Her2*^*t/w*^*/Adamts18*^+/+^ and *Her2*^*t/w*^*/Adamts18*^−/−^ mammary glands (data not shown).

### Low *ADAMTS18* expression in distant metastases of HER2 + tumors relapsed posttrastuzumab treatment

To investigate the clinical association of *ADAMTS18* with HER2 + breast cancer, GEO dataset (GSE191230) was used for the analysis (Additional file 1: Table S4). In this dataset, RNA sequencing (RNA-seq) analysis was performed on 13 treatment-naive HER2 + breast tumors and 7 distant metastases of HER2 + tumors relapsed posttrastuzumab treatment (Additional file 1: Fig. S6A). *ADAMTS18* expression was significantly downregulated in distant metastatic samples compared with the primary tumors (Additional file 1: Fig. S6B).

## Discussion

**Fig. 8:**
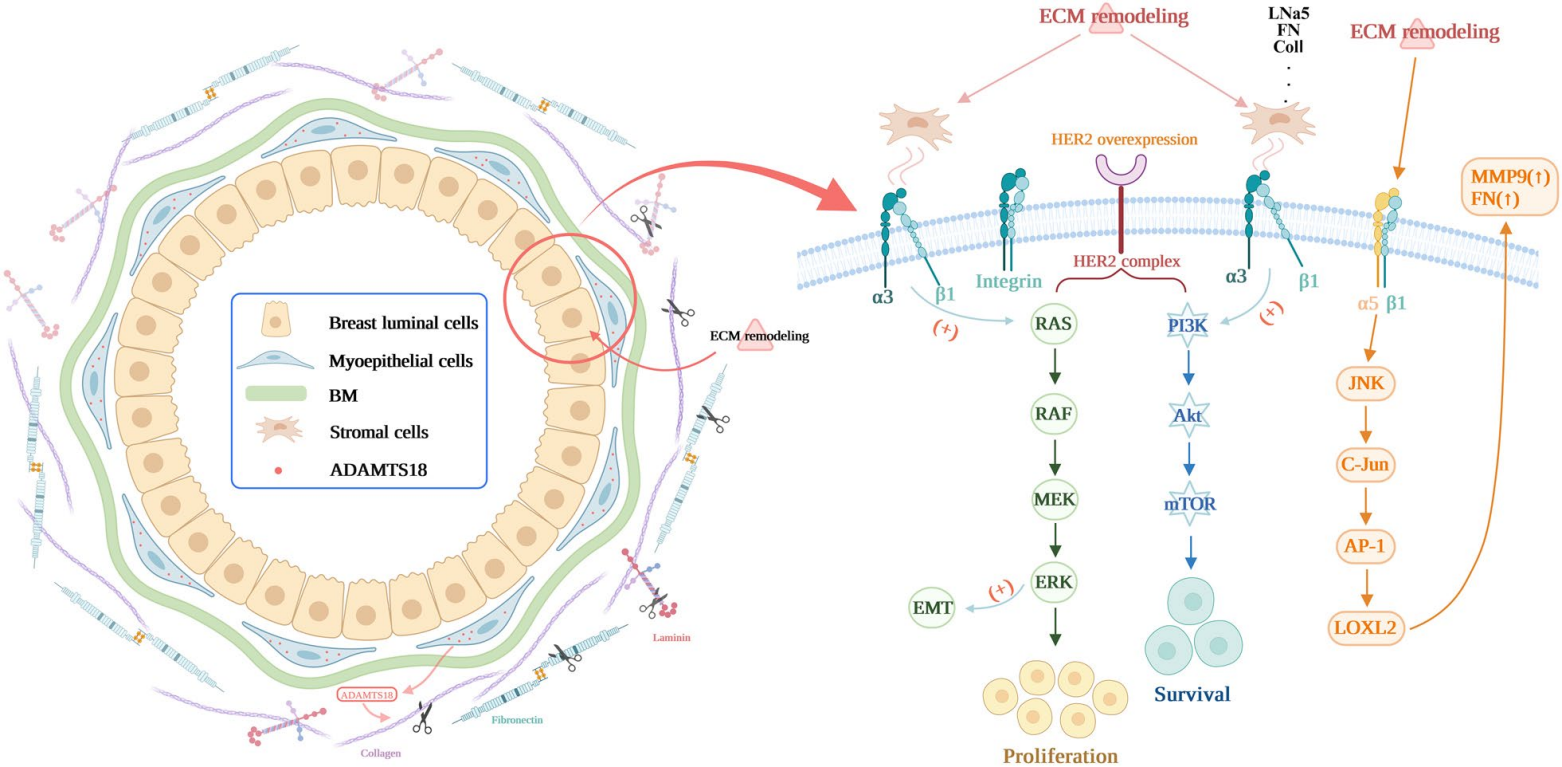
Working model of ADAMTS18 in HER2-positive mammary tumorigenesis and metastasis. ADAMTS18 is secreted by myoepithelial cells of mammary ducts and functions as an important regulator that links basement membrane (BM) and stroma extracellular matrix (ECM) alterations to HER2-positive tumor behavior. ADAMTS18 deficiency results in aberrant expression levels and distribution of some ECM molecules (e.g. LNα5, FN, and ColI) in BM and stroma, resulting in enhanced activity of integrin (Itgα3/α5/β1)-mediated PI3K/AKT, ERK, and JNK signaling activity. These alterations may increase the risk of HER2-positive mammary tumorigenesis and metastasis. (Cartoon picture was created with BioRender.com)

In this study, we conducted a long-term study with a large cohort of genetically modified mice and demonstrated that ADAMTS18 deficiency associates extracellular matrix dysfunction with a higher risk of HER2-positive mammary tumorigenesis and metastasis (Fig. 8). To our knowledge, this is the first in vivo evidence demonstrating the association between ADAMTS metalloproteinase and mammary tumor progression from the perspective of extracellular matrix remodeling.

We demonstrated that *Adamts18* gene knockout (*Adamts18*^*−/−*^) resulted in a significantly increased incidence of spontaneous mammary tumors and metastases in heterozygous *Her2* transgenic (*Her2*^*t/w*^) mice (Fig. 2). This was further supported by the finding that the proliferation, migration and invasion capacity of primary *Her2*^*t/w*^*/Adamts18*^−/−^ mammary tumor cells were significantly higher than that of primary *Her2*^*t/w*^*/Adamts18*^+/+^ mammary tumor cells cultured in vitro (Fig. 3). In this work, we did not observe the effect of ADAMTS18 in homozygous *Her2* transgenic (*Her2*^*t/t*^) mice for the following reason. Since  almost all *Her2*^*t/t*^ mice develop mammary tumor and metastasis in a relatively short period of time [24, 25], it is difficult to detect differences in the incidence of mammary tumor and metastasis between *Adamts18* gene knockout (*Adamts18*^*−/−*^) mice and the wildtype control (*Adamts18*^+*/*+^) mice. Compared with homozygous *Her2*^*t/t*^ mice, heterozygous *Her2*^*t/w*^ mice had significantly lower incidence of mammary tumors and longer average tumor latency, which may be more helpful for observing the synergistic effect of *Adamts18* gene knockout on the process of mammary tumorigenesis and malignant progression in HER2-overexpressing mice. In this work, 30-month-old *Her2*^*t/w*^*/Adamts18*^−/−^ mice exhibited significantly increased number of Ki-67-positive ductal epithelial cells (Fig. 4). This phenotype was observed as early as 10 months of age in *Her2*^*t/w*^*/Adamts18*^−/−^ mice, suggesting that ADAMTS18 deficiency promotes mammary hyperplasia. The underlying mechanism could be partially attributed to increased activity of PI3K/AKT, ERK, JNK signaling pathway and potentiated EMT in *Her2*^*t/w*^*/Adamts18*^−/−^ mice.

Numerous studies have shown that the ECM plays a critical role in breast tumorigenesis [4–6]. In the mammary glands, ECM proteins are mainly synthesized and secreted by myoepithelial cells. ADAMTS18 is also secreted by myoepithelial cells and functions as an ECM modifier. We thus sought to establish the association between ECM alterations caused by ADAMTS18 deficiency and tumor behavior. Our data clearly show that ADAMTS18 deficiency causes changes of mammary ECM molecules (e.g., LNα5, FN, ColI). LN is the main component of the basement membrane, consisting of α, β and γ chains [29, 30]. There are at least 16 laminin isoforms that have been described in mammal tissues, and each of which exhibits unique spatio-temporal expression patterns and biological functions. Among them, LN-511 is a potent adhesive and pro-migration substrate that has been shown to promote haptotactic migration of human and mouse carcinoma cells by binding to cell surface integrin receptors such as α3β1, α6β1, α7β1, and α6β4 [31, 32]. Binding of LN-511 to integrin receptors triggers the formation of FAK, PI3K, ERK, and RhoA signaling pathways that drive tumor cell migration and invasion [33–35]. Our data showed that the mammary glands of 30-month-old *Her2*^t/w^*/Adamts18*^−/−^ mice have significantly elevated levels of LNα5, ITGα3, α5, β1, p-ERK, and p-AKT. Activation of AKT pathway promotes EMT, which plays a crucial role in breast cancer metastasis [36]. The expression profiles of E-cadherin, N-cadherin, and FN in mammary glands of *Her2*^*t/w*^*/Adamts18*^−/−^ mice suggest the involvement of EMT process.

Increased synthesis and deposition of FN in human breast cancer tissues has been shown to be associated with poor clinical prognosis [37–39]. In this study, we found that FN levels were significantly increased in mammary glands of 30-month-old *Her2*^*t/w*^*/Adamts18*^−/−^ mice. This may be due to the following reasons. First, FN is a potential substrate for ADAMTS18. ADAMTS18 has been shown to cleave recombinant FN at the N-terminal linker sequence between domain (I)5 and (I)6 [15], which is similar to its homolog ADAMTS16 or other proteases such as ADAMTS2, ADAMTS3, and MMP3 [40–43]. This action may indirectly affect the homeostasis of other ECM molecules, such as collagen and laminin [15]. Elevated FN levels in the mammary glands of *Her2*^*t/w*^*/Adamts18*^−/−^ mice may be caused by reduced proteolytic cleavage of ADAMTS18. Second, FN is one of the important indicators involved in EMT process. Its effect on EMT is mediated by the ERK kinase pathway [44]. Our data showed enhanced ERK activity and EMT processes in the mammary glands of 30-month-old *Her2*^*t/w*^*/Adamts18*^−/−^ mice, suggesting increased *Fn* transcription. We also observed that FN was mainly distributed in *Her2*^*t/w*^*/Adamts18*^−/−^ BM. It has been reported that the deposition of FN in the basement membrane is related to lymphatic metastasis of breast cancer [45].

Provenzano et al. found that in breast cancer ColI levels increased and ColIV levels decreased due to degradation of the basement membrane, which is associated with a higher risk of invasion and malignancy [46]. In this study, elevated ColI levels in *Her2*^*t/w*^*/Adamts18*^−/−^ mammary glands suggested an increased risk of mammary tumor metastasis. In addition, increased activation of ITGα5β1/JNK/c-JUN signaling pathway and elevated levels of LOXL2 were also observed in the mammary glands of 30-month-old *Her2*^*t/w*^*/Adamts18*^−/−^ mice. Activation of these signals has been shown to induce fibroblasts to produce FN and MMP9 [47]. Indeed, a significant increase in MMP9 activity was detected in the mammary glands of *Her2*^*t/w*^*/Adamts18*^−/−^ mice, which may disrupt basement membrane integrity and increase the risk of metastasis.

Aberrant activation of PI3K/AKT is thought to be responsible for the development of trastuzumab resistance [1, 3]. Our data showed that ADAMTS18 deficiency in the mammary glands of 30-month-old mice significantly increases the activity of PI3K/AKT signaling pathway. Moreover, in samples of HER2 + tumors with distant metastases that recurred after trastuzumab treatment, *ADAMTS18* expression levels were significantly lower than those in treatment-naive HER2 + primary breast tumors. These findings suggest that ADAMTS18 may be used as a reference factor for drug selection in patients with trastuzumab resistance.

Previously, Ataca et al. reported that ADAMTS18 deficiency alters the mammary stem cell niche through remodeling BM and/or stroma ECM molecules, thereby reducing the regeneration of mammary epithelial cells [15]. According to this view, *Adamts18*^−/−^ mice should have fewer mammary epithelial cells than their WT littermates, thus *Adamts18*^−/−^ mammary glands should be less prone to oncogenic transformation. Inconsistent with this assumption, we found that ADAMTS18 deficiency promotes HER2-positive mammary tumorigenesis and metastasis. The discrepancy remains unclear but may be related to the following factors. The previous studies focused on mammary development during puberty. At the stage of puberty, estrogen and progesterone levels are relatively high and induce ADAMTS18 production in myoepithelial cells via typical Wnt signaling, which helps to remodel BM and/or stroma ECM for optimal stem cell regenerative capacity. In the present study, we focused on *Her2*^*t/w*^*/Adamts18*^−/−^ and *Her2*^*t/w*^*/Adamts18*^+/+^ mice at 30 months of age. At this age, the expression levels of estrogen and progesterone are significantly lower than those during puberty. Importantly, the HER2 proto-oncogene was introduced in these mice, and the proliferation and metastasis of the mouse mammary glands were primarily driven by HER2-related signals.

The main limitations of this study are as follows: (1) The interpretation of these findings remains limited and incomplete because of the systemic deletion of *Adamts18* gene and the inability to exclude that changes in other organs (for instance the immune system) might contribute to the phenotypes observed. (2) The exact substrates of ADAMTS18 in HER2-positive mammary tumorigenesis and metastasis are still unknown. They may be the ECM molecules observed in this study (e.g., FN), or they may be some undiscovered cytokines, such as VEGF or TGFβ or their precursors. Thus, we cannot rule out that other signaling pathways (e.g., VEGF or TGFβ signaling pathway), may also be involved in ADAMTS18-mediated mammary tumor progression. (3) Based on the current model, the role of ADAMTS18 in other types of breast cancer, such as estrogen-related luminal A/B type, basal-like type, and myoepithelioma, remains unclear and requires further study. In addition, we did not consider the effect of ADAMTS18 on mammary tumorigenesis and metastasis during pregnancy. Whether pregnancy promotes ADAMTS18 secretion and thus affects the development of breast cancer remains to be clarified in future studies. (4) The association of ADAMTS18 with HER2-positive breast cancer still needs to be verified in a large number of clinical samples.

## Conclusions

In summary, this study suggests that ADAMTS18 deficiency associates mammary ECM dysfunction with a higher risk of HER2-positive mammary tumorigenesis and metastasis.

### Supplementary Information

**Additional file 1.** Supplemental information file. **Figure S1.** Generation and characterization of Her2t/w/Adamts18^+/+^ mice and Her2t/w/Adamts18^−/−^ mice with C57BL/6-FVB mixed background. (A) The breeding strategy of Her2t/w transgenic mouse model in the presence or absence of ADAMTS18 was described in method. Her2t/w/Adamts18^+/+^ mice (n = 36); Her2t/w/Adamts18^−/−^ mice (n = 32). (B) Monitoring of mouse body weight during the period of observation. (C) The survival rate of mice until 30-month-old. P values were determined through Log-rank (Mantel-Cox) test. **Figure S2.** Average tumor numbers in tumor-bearing Her2t/w/Adamts18^+/+^ mice and Her2t/w/Adamts18^−/−^ mice. Each dot or square represents an individual. **Figure S3.** Representative images and HE staining of metastatic tumors in the peritoneal cavity, liver, and kidney of Her2t/w/Adamts18^−/−^ mice. Scale bars, 5 mm (left panel) and 100 μm (right panel). **Figure S4.** ADAMTS18 deficiency induces early proliferation of mammary epithelial cells (A) Representative immunohistochemical staining of cross-sections of mammary glands from the indicated genotypes using Ki-67 antibody. The red arrows are Ki-67 positive signals that were detected in Her2t/w/Adamts18^−/−^ mice at 10 months of age. Scale bars = 100 μm. (B) Quantification of Ki-67-positive cells (%) (n = 4/group). Data are expressed as mean ± SD. **p < 0.01; Student’s t-test, two tailed. **Figure S5.** Enhanced ERK1/2 and PI3K/AKT signaling activity and epithelial-mesenchymal transitions (EMT) process in mammary glands of 30-month-old Her2t/w/Adamts18^−/−^ mice. (A) Western blot analysis of the protein levels of total ERK1/2 (t-ERK1/2), phosphorylated ERK1/2 (p-ERK1/2), AKT, phosphorylated AKT, E-cadherin, Ncadherin, and fibronectin in mammary glands of 30-month-old virgin Her2t/w/Adamts18^+/+^ and Her2t/w/Adamts18^−/−^ mice. (B-F) Relative expression levels of the proteins are represented as p-ERK1/2/t-ERK1/2, p-AKT/AKT or protein/GAPDH. Each dot or square represents an individual. Data are expressed as means ± SD (n = 4). *p < 0.05; **p < 0.01; ***p < 0.001; Student’s t-test, two tailed. **Figure S6.** Low ADAMTS18 expression in distant metastases of HER2+ tumors relapsed posttrastuzumab treatment. (A) HER2-positive breast cancer-associated GEO dataset (GSE191230). In this dataset, RNA sequencing (RNA-seq) analysis was performed on 13 treatment-naïve HER2+ breast tumors (n = 13) and 7 distant metastases of HER2+ tumors relapsed posttrastuzumab treatment (n = 7), including 2 lung metastases and 5 brain metastases. (B) Analysis of ADAMTS18 expression. RNA expression was quantified with transcripts per kilobase of exon model per million mapped reads (TPM). Data are expressed as mean ± SD. *p < 0.05; two-tailed Student’s ttest. **Table S1.** Antibodies used in this study. **Table S2.** Total tumor burden at endpoint. **Table S3.** Determination of mRNA levels of main ECM molecules in BM and stroma as well as the mammary epithelial cell receptors by qRT-PCR. **Table S4.** The sample information of HER2-positive breast cancer patients.

## Data Availability

Requests for resources, reagents, and further information will be made available from the lead corresponding author (Wei Zhang) on reasonable request.
