# Supplementary material for: ADAMTS18 deficiency associates extracellular matrix dysfunction with a higher risk of HER2-positive mammary tumorigenesis and metastasis
Source: Breast Cancer Res. 2024 Jan 29;26:19. doi: 10.1186/s13058-024-01771-3 (PMC10826190; doi:10.1186/s13058-024-01771-3)
Supplement: Supplementary file 1 — Additional file 1. Supplemental information file. Figure S1. Generation and characterization of Her2t/w/Adamts18+/+ mice and Her2t/w/Adamts18−/− mice with C57BL/6-FVB mixed background. (A) The breeding strategy of Her2t/w transgenic mouse model in the presence or absence of ADAMTS18 was described in method. Her2t/w/Adamts18+/+ mice (n = 36); Her2t/w/Adamts18−/− mice (n = 32). (B) Monitoring of mouse body weight during the period of observation. (C) The survival rate of mice until 30-month-old. P values were determined through Log-rank (Mantel-Cox) test. Figure S2. Average tumor numbers in tumor-bearing Her2t/w/Adamts18+/+ mice and Her2t/w/Adamts18−/− mice. Each dot or square represents an individual. Figure S3. Representative images and HE staining of metastatic tumors in the peritoneal cavity, liver, and kidney of Her2t/w/Adamts18−/− mice. Scale bars, 5 mm (left panel) and 100 μm (right panel). Figure S4. ADAMTS18 deficiency induces early proliferation of mammary epithelial cells (A) Representative immunohistochemical staining of cross-sections of mammary glands from the indicated genotypes using Ki-67 antibody. The red arrows are Ki-67 positive signals that were detected in Her2t/w/Adamts18−/− mice at 10 months of age. Scale bars = 100 μm. (B) Quantification of Ki-67-positive cells (%) (n = 4/group). Data are expressed as mean ± SD. **p < 0.01; Student’s t-test, two tailed. Figure S5. Enhanced ERK1/2 and PI3K/AKT signaling activity and epithelial-mesenchymal transitions (EMT) process in mammary glands of 30-month-old Her2t/w/Adamts18−/− mice. (A) Western blot analysis of the protein levels of total ERK1/2 (t-ERK1/2), phosphorylated ERK1/2 (p-ERK1/2), AKT, phosphorylated AKT, E-cadherin, Ncadherin, and fibronectin in mammary glands of 30-month-old virgin Her2t/w/Adamts18+/+ and Her2t/w/Adamts18−/− mice. (B-F) Relative expression levels of the proteins are represented as p-ERK1/2/t-ERK1/2, p-AKT/AKT or protein/GAPDH. Each dot or square represents an individual. Data [file 13058_2024_1771_MOESM1_ESM.docx]

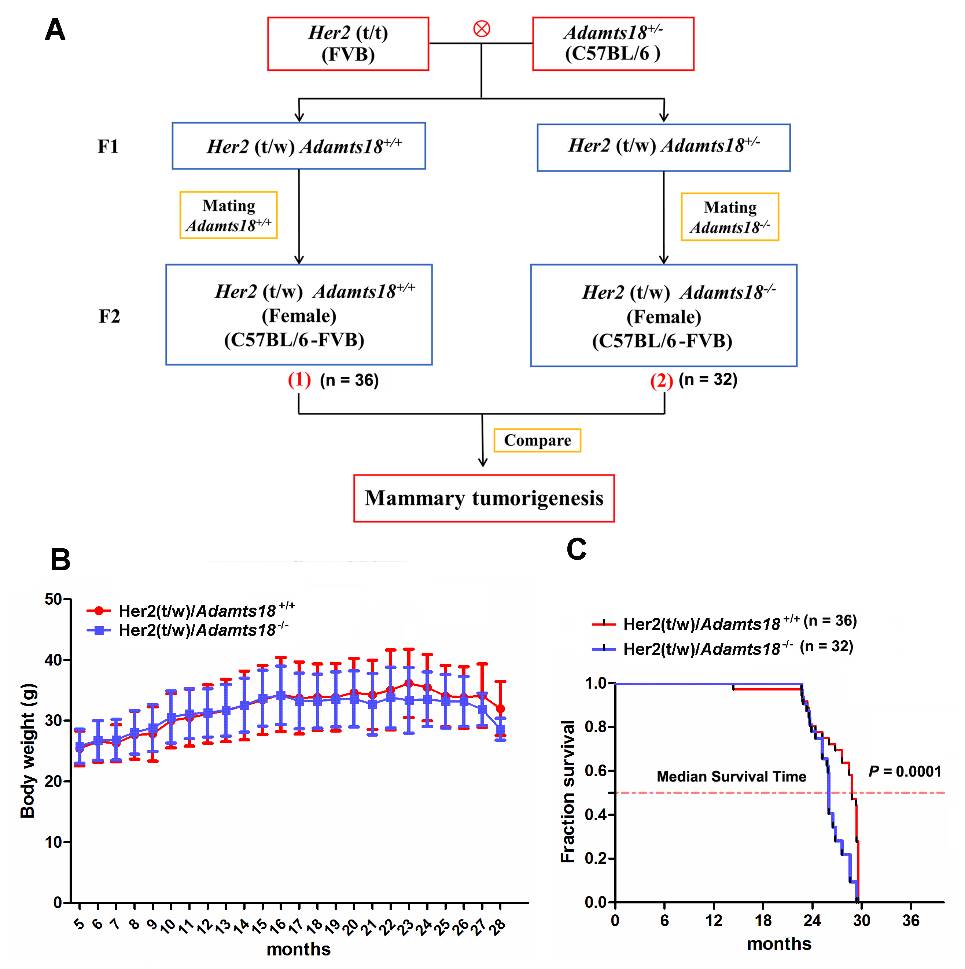


**Figure S1.** Generation and characterization of *Her2^t/w^/Adamts18^+/+^* mice and *Her2^t/w^/Adamts18^-/-^* mice with C57BL/6-FVB mixed background. **(A)** The breeding strategy of *Her2^t/w^* transgenic mouse model in the presence or absence of ADAMTS18 was described in method. *Her2^t/w^/Adamts18^+/+^* mice (n = 36); *Her2t/w/Adamts18^-/-^* mice (n = 32). **(B)** Monitoring of mouse body weight during the period of observation. **(C)** The survival rate of mice until 30-month-old. *P* values were determined through Log-rank (Mantel-Cox) test.


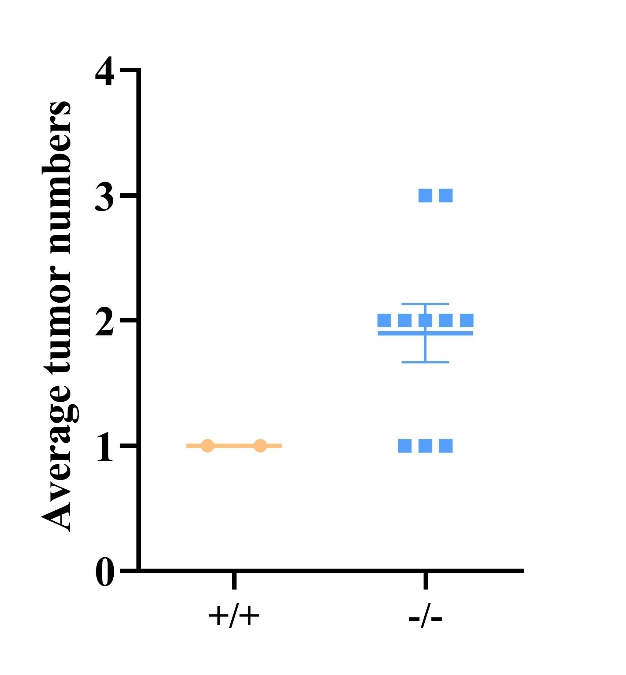


**Figure S2.** Average tumor numbers in tumor-bearing *Her2^t/w^/Adamts18*^+/+^ mice and *Her2*^t/w^*/Adamts18*^-/-^ mice. Each dot or square represents an individual.

**
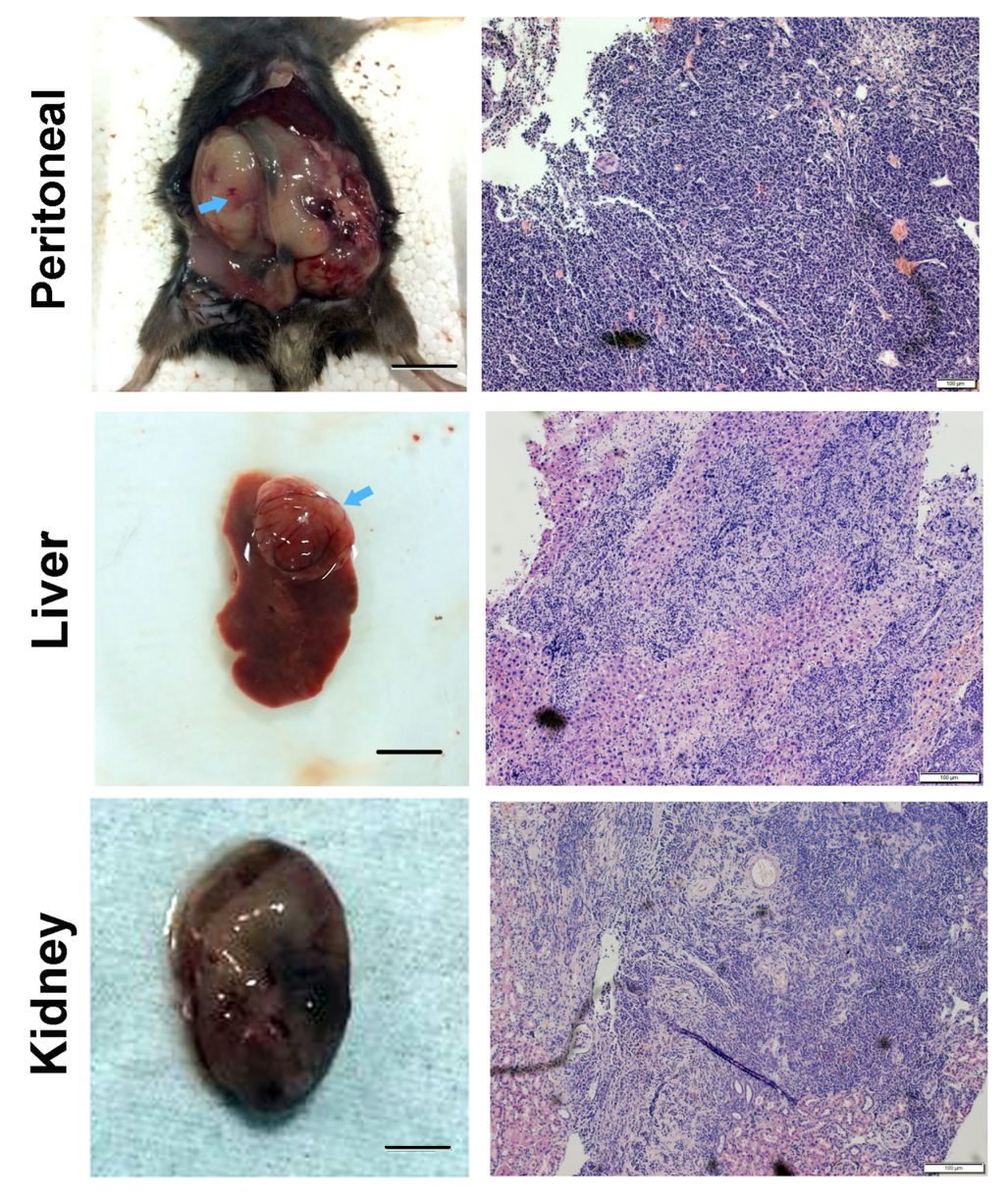
**

**Figure S3.** Representative images and HE staining of metastatic tumors in the peritoneal cavity, liver, and kidney of *Her2^t/w^/Adamts18*^-/-^ mice. Scale bars, 5 mm (left panel) and 100 μm (right panel).


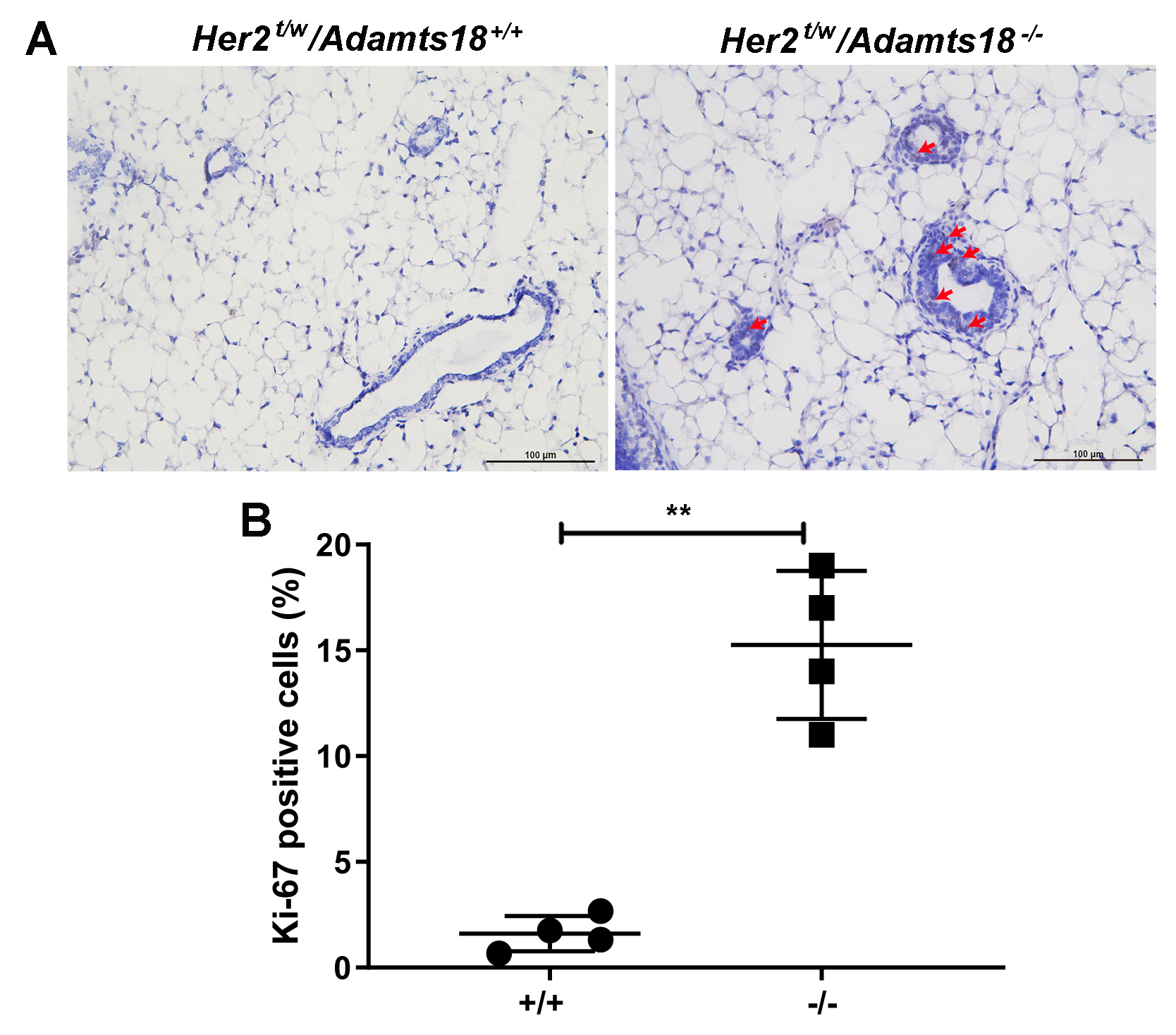


**Figure S4.** ADAMTS18 deficiency induces early proliferation of mammary epithelial cells **(A)** Representative immunohistochemical staining of cross-sections of mammary glands from the indicated genotypes using Ki-67 antibody. The red arrows are Ki-67 positive signals that were detected in *Her2^t/w^/Adamts18^-/^*^-^ mice at 10 months of age. Scale bars = 100 μm. **(B)** Quantification of Ki-67-positive cells (%) (n = 4/group). Data are expressed as mean ± SD. ***p* < 0.01; Student’s *t*-test, two tailed.

**
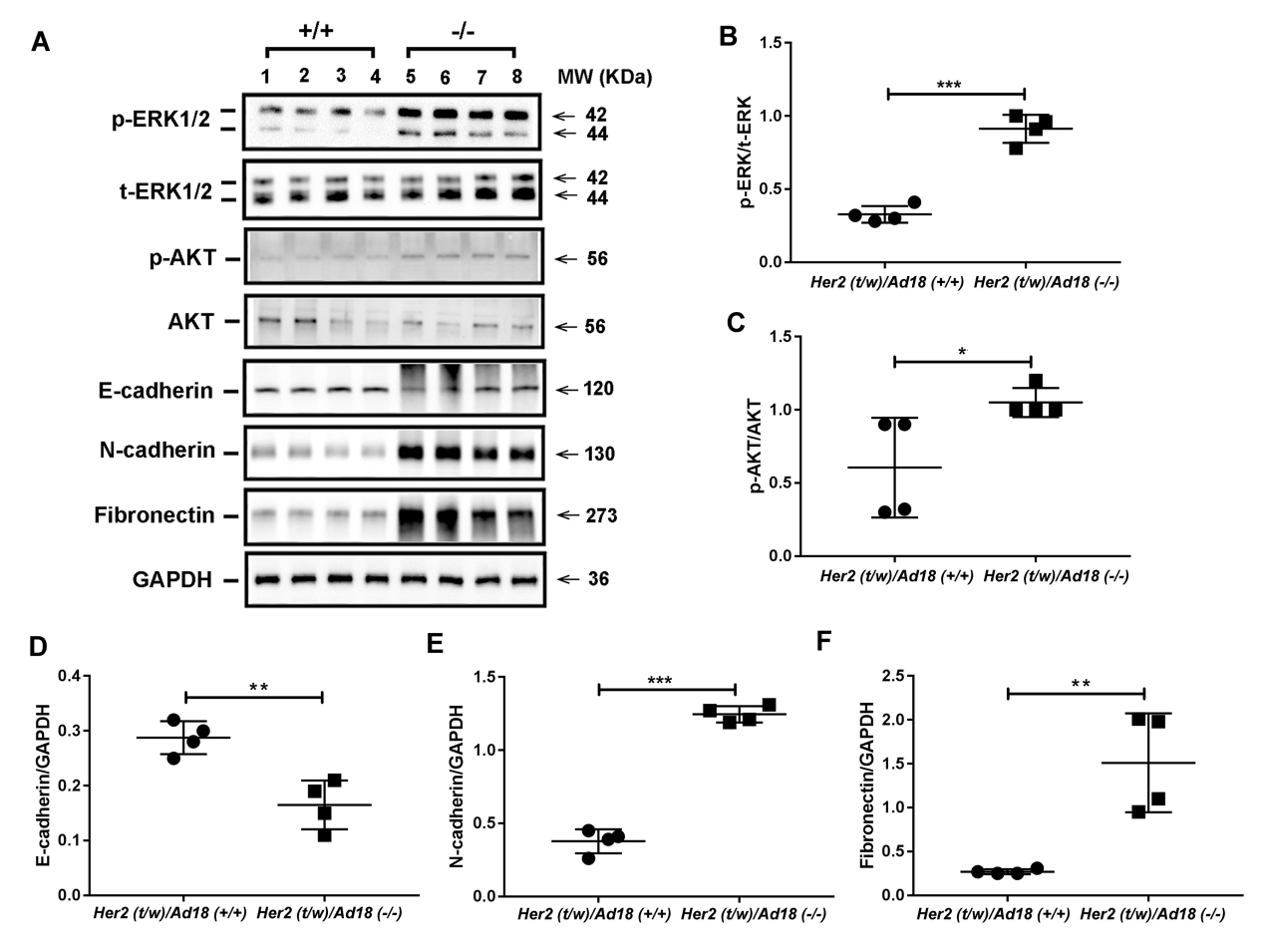
**

**Figure S5.** Enhanced ERK1/2 and PI3K/AKT signaling activity and epithelial-mesenchymal transitions (EMT) process in mammary glands of 30-month-old *Her2^t/w^/Adamts18*^-/-^ mice. **(A)** Western blot analysis of the protein levels of total ERK1/2 (t-ERK1/2), phosphorylated ERK1/2 (p-ERK1/2), AKT, phosphorylated AKT, E-cadherin, N-cadherin, and fibronectin in mammary glands of 30-month-old virgin *Her2^t/w^/Adamts18*^+/+^ and *Her2^t/w^/Adamts18*^-/-^ mice. **(B-F)** Relative expression levels of the proteins are represented as p-ERK1/2/t-ERK1/2, p-AKT/AKT or protein/GAPDH. Each dot or square represents an individual. Data are expressed as means ± SD (n = 4). **p* < 0.05; ***p* < 0.01; ****p* < 0.001; Student’s *t*-test, two tailed.


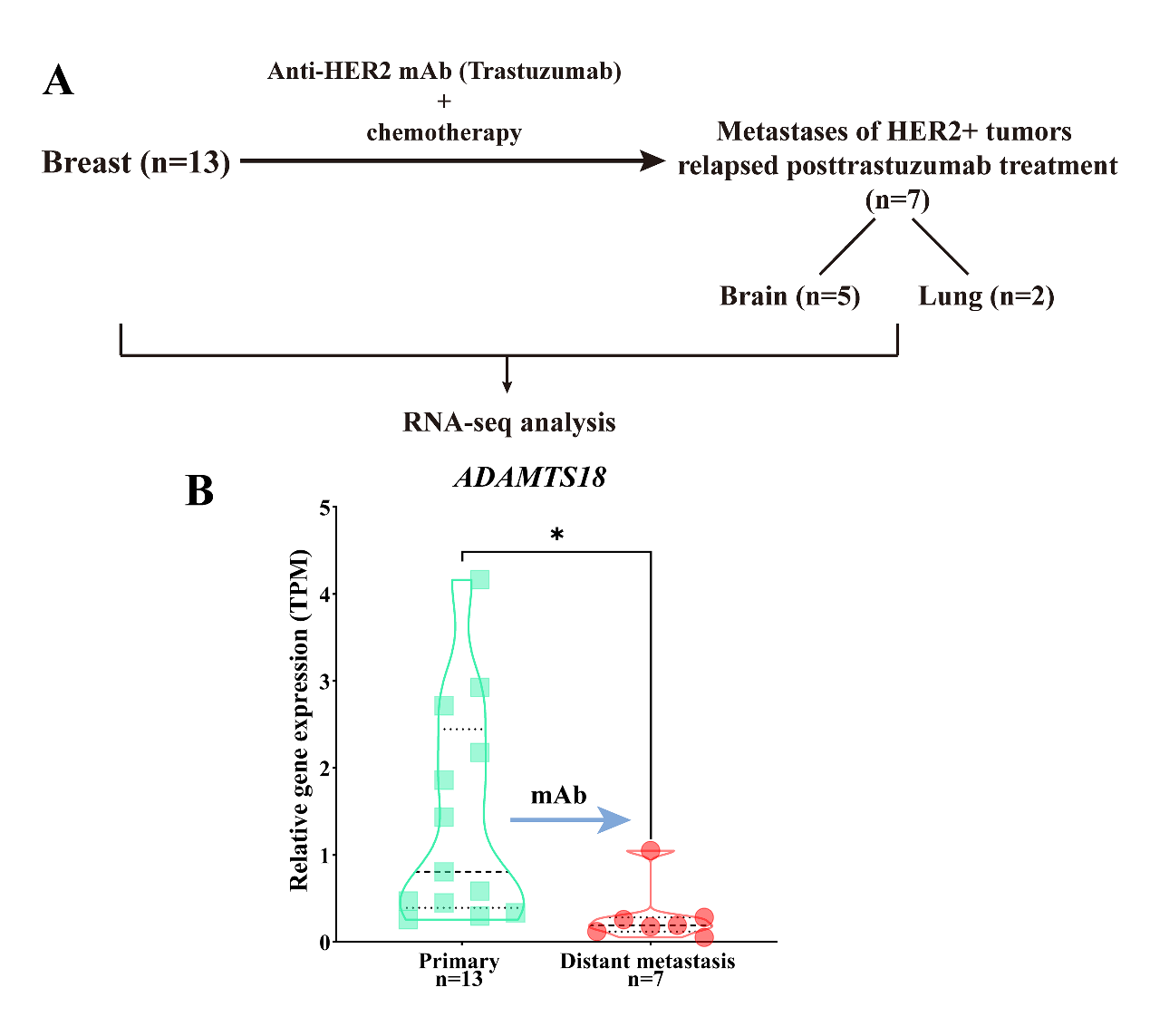


**Figure S6.** Low *ADAMTS18* expression in distant metastases of HER2+ tumors relapsed posttrastuzumab treatment. **(A)** HER2-positive breast cancer-associated GEO dataset (**GSE191230**). In this dataset, RNA sequencing (RNA-seq) analysis was performed on 13 treatment-naïve HER2+ breast tumors (n = 13) and 7 distant metastases of HER2+ tumors relapsed posttrastuzumab treatment (n = 7), including 2 lung metastases and 5 brain metastases. **(B)** Analysis of *ADAMTS18* expression. RNA expression was quantified with transcripts per kilobase of exon model per million mapped reads (TPM). Data are expressed as mean ± SD. **p* < 0.05; two-tailed Student’s t-test.

**Table S1.** Antibodies used in this study.

| **Name** | **Catalog**  **number** | **Company** | **Application**  **(dilution)** |
| --- | --- | --- | --- |
| Anti-p44/42 MAPK (Erk1/2) (137F5) | 4695T | Cell Signaling Technology | WB (1:1000)  IHC-P (1:300) |
| Anti-Phospho-p44/42 MAPK (Erk1/2) (Thr202/Tyr204) | 9101S | Cell Signaling Technology | WB (1:1000) |
| Anti-Phospho-p44/42 MAPK (Erk1/2) (Thr202/Tyr204) (D13.14.4E) XP | 4370T | Cell Signaling Technology | IHC-P (1:500) |
| Anti-AKT | 9272 | Cell Signaling Technology | WB (1:1000) |
| Anti-Phospho-AKT(Ser473) | 9271 | Cell Signaling Technology | WB (1:1000) |
| Anti E-Cadherin | 610181 | BD Biosciences | WB (1:2500) |
| Anti-N-cadherin | 66219-1-Ig | Proteintech | WB (1:2000) |
| Anti-Laminin alpha 5 | NBP1-18714 | Novus Biologicals | WB (1:1000) |
| Anti-Loxl2 | ab96233 | abcam | WB (1:500) |
| Anti-Fibronectin | ab199056 | abcam | IHC-P (1:2000)  WB (1:1000) |
| Anti-Laminin | ab11575 | abcam | IHC (1:200) |
| Anti-Collagen I | ab270993 | abcam | IHC (1:100) |
| Anti-GAPDH | AB0037 | Abways | WB (1:3000) |
| Anti-ITGA3 | ab223661 | abcam | WB (1:1000) |
| Anti-ITGA5 | Ab150361 | abcam | WB (1:1000) |
| Anti-ITGB1 | Ab179471 | abcam | WB (1:2000) |
| Anti-MMP9 (N-terminal) | 10375-2-AP | Proteintech | WB (1:1000) |
| Anti-c-JUN | ab40766 | abcam | WB (1:1000) |
| Anti-Phospho-c-JUN (S63) | ab32385 | abcam | WB (1:1000) |

**Table S2.** Total tumor burden at endpoint.

| **Mouse** | **Mammary** | **Lung** | **Liver** | **Kidney** | **Peritoneal** |
| --- | --- | --- | --- | --- | --- |
| 1(+/+) | + | - | - | - | - |
| 2(+/+) | + | - | - | - | - |
| 1(-/-) | + | + | - | + | + |
| 2(-/-) | + | + | + | + | + |
| 3(-/-) | + | + | - | - | - |
| 4(-/-) | + | + | + | + | + |
| 5(-/-) | + | - | - | + | - |
| 6(-/-) | + | + | + | - | - |
| 7(-/-) | + | + | + | - | - |
| 8(-/-) | + | + | - | + | - |
| 9(-/-) | + | + | + | - | - |
| 10(-/-) | + | + | + | - | - |

(+/+), *Her2^t/w^/Adamts18*^+/+^ mice; (-/-), *Her2^t/w^/Adamts18*^-/-^ mice

**Table S3.** Determination of mRNA levels of main ECM molecules in BM and stroma as well as the mammary epithelial cell receptors by qRT-PCR.

| **Mammary glands** | **Gene Name** | **Protein Description** | ***Her2 ^t/w^/Adamts18*^+/+^**  **(n = 3)** | ***Her2^t/w^/Adamts18*^-/-^** **(n = 3)** | ***P* value** |
| --- | --- | --- | --- | --- | --- |
| **Basement Membrane and Stroma** | *Lama1* | Laminin alpha1 | 1.007 ± 0.191 | 1.067 ± 0.123 | 0.8042 |
|  | *Lama5* | Laminin alpha5 | 0.694 ± 0.07 | 1.041 ± 0.056 | 0.048* |
|  | *Lamb1* | Laminin beta 1 | 0.7633 ± 0.1715 | 1.157 ± 0.2413 | 0.2547 |
|  | *Lamb2* | Laminin beta 2 | 0.8282 ± 0.1718 | 0.8688 ± 0.1149 | 0.7504 |
|  | *Lamb3* | Laminin beta 3 | 0.7401 ± 0.2599 | 0.5754 ± 0.1597 | 0.7016 |
|  | *Lamc1* | Laminin gamma 1 | 0.977 ± 0.153 | 1.173 ± 0.127 | 0.3789 |
|  | *Lamc2* | Laminin gamma 2 | 0.7936 ± 0.2064 | 0.6387 ± 0.1193 | 0.6672 |
|  | *FN1* | Fibronectin | 0.7389 ± 0.0362 | 0.9728 ± 0.0262 | 0.0064** |
|  | *Col1a1* | Collagen alpha-1 chain | 0.7558 ± 0.0292 | 1.064 ± 0.059 | 0.01** |
|  | *Nid1* | Nidogen 1 | 0.6096 ± 0.3904 | 0.3893 ± 0.1079 | 0.3434 |
|  | *Vcan* | Versican | 0.6016 ± 0.3984 | 0.5568±0.3095 | 0.8410 |
| **Epithelial Cell Receptor** | *Itga3* | Integrin alpha 3 | 0.6238 ± 0.041 | 0.91 ± 0.0614 | 0.018* |
|  | *Itga5* | Integrin alpha 5 | 0.8699 ± 0.0524 | 1.051 ± 0.035 | 0.045* |
|  | *Itgb1* | Integrin beta 1 | 0.7837 ± 0.0591 | 0.9634 ± 0.025 | 0.049* |
|  | *Itgb3* | Integrin beta 3 | 1.795±0.7947 | 2.274±0.2108 | 0.3301 |
|  | *Dag1* | Dystroglycan 1 | 0.7305±0.2695 | 0.6091±0.1010 | 0.4567 |
|  | *Ddr1* | Discoidin domain receptor family, member 1 | 0.8291±0.1709 | 0.5624±0.06215 | 0.4442 |

^*^represents *P* ≤ 0.05; ^**^represents *P* ≤ 0.01.

**Table S4.** The sample information of HER2-positive breast cancer patients.

| **Sample title** | **Sample organism** | **Sample characteristics** |
| --- | --- | --- |
| Metastatic tumor_1 | Homo sapiens | Breast Cancer Metastasis to Lung |
| Metastatic tumor_2 | Homo sapiens | Breast Cancer Metastasis to Brain |
| Metastatic tumor_3 | Homo sapiens | Breast Cancer Metastasis to Lung |
| Metastatic tumor_4 | Homo sapiens | Breast Cancer Metastasis to Brain |
| Metastatic tumor_5 | Homo sapiens | Breast Cancer Metastasis to Brain |
| Metastatic tumor_6 | Homo sapiens | Breast Cancer Metastasis to Brain |
| Metastatic tumor_7 | Homo sapiens | Breast Cancer Metastasis to Brain |
| Primary tumor_1 | Homo sapiens | Primary tumor |
| Primary tumor_2 | Homo sapiens | Primary tumor |
| Primary tumor_3 | Homo sapiens | Primary tumor |
| Primary tumor_4 | Homo sapiens | Primary tumor |
| Primary tumor_5 | Homo sapiens | Primary tumor |
| Primary tumor_6 | Homo sapiens | Primary tumor |
| Primary tumor_7 | Homo sapiens | Primary tumor |
| Primary tumor_8 | Homo sapiens | Primary tumor |
| Primary tumor_9 | Homo sapiens | Primary tumor |
| Primary tumor_10 | Homo sapiens | Primary tumor |
| Primary tumor_11 | Homo sapiens | Primary tumor |
| Primary tumor_12 | Homo sapiens | Primary tumor |
| Primary tumor_13 | Homo sapiens | Primary tumor |
